# Roles of maize *WAK* gene family in responses to abiotic and biotic stresses, and hormonal treatments

**DOI:** 10.3389/fpls.2025.1652811

**Published:** 2025-09-16

**Authors:** Xiangnan Li, Yuwei Bi, Haoye Feng, Yanming Cai, Hang Chen, Peisen Su, Yong Song, Nan Li, Yinglun Fan, Like Liu, Lingzhi Meng, Chunmei Zong

**Affiliations:** ^1^ College of Agriculture and Biology, Liaocheng University, Liaocheng, China; ^2^ Mudanjiang Branch of Heilongjiang Academy of Agricultural Sciences, Mudanjiang, China

**Keywords:** maize (*Zea mays* L.), WAK gene family, abiotic and biotic stresses, hormonal treatments, expression pattern analysis

## Abstract

**Introduction:**

Wall-associated receptor kinases (WAKs) are a family of receptor-like kinases (RLKs) that play important roles in the communication between the plant cell wall and the cytoplasm. *WAKs* have been identified in several plants. However, a comprehensive investigation of maize WAKs has not been performed yet.

**Methods:**

In this study, the maize *WAK* gene family was identified through whole-genome scanning. y -30The physicochemical characteristics, chromosomal locations, phylogenetic tree, gene structures, conserved motifs, gene duplication, collinearity, and cis-acting elements of maize *WAKs* were analyzed.

**Results:**

A total of 56 *ZmWAKs* were identified in the maize genome and divided into seven subgroups. Among these, 54 genes were successfully mapped to maize chromosomes. Gene duplication events were detected in 13 *ZmWAKs*, with nine segmental (SD) and two tandem duplication (TD) events. Maize *WAKs* exhibited zero, eight, 27, and 41 collinear links with the *WAKs* from *Arabidopsis*, soybean, rice, and sorghum, respectively. In the promoter regions of *ZmWAKs*, a total of 107 types of cis-acting elements were predicted. Among them, the functions of 82 elements are known. These elements are associated with plant growth and development and light, hormones, stress, and defense responses. The transcriptome data analysis showed that *ZmWAKs* displayed tissue-specific expression and are involved in the responses to various abiotic and biotic stresses, including cold, salt, drought, waterlogging, pathogens, and pests. *ZmWAK9, ZmWAK15, ZmWAK27, ZmWAK41*, and *ZmWAK49* are significantly induced by multiple stress conditions, indicating their crucial roles in stress responses and potential value for further research.

**Discussion:**

Our results provide insights into the function of maize *WAKs* in response to abiotic and biotic stresses and offer a theoretical foundation for understanding their mechanisms of action.

## 1 Introduction

Plant receptor-like kinases (RLKs) are a subgroup of serine/threonine (Ser/Thr) kinases and play a crucial role in plant growth, development, and stress responses (Shiu and Bleecker, 2001). The RLK family is a large protein family in plants. It is divided into different subfamilies based on the extracellular domains and the kinase domains, including cysteine-rich receptor-like kinases (CRKs), lysin motif receptor-like kinases (LysM-RLKs), cell wall-associated receptor kinases (WAKs), and leucine-rich receptor-like kinases (LRR-RLKs), etc. (Yu et al., 2025; Abedi et al., 2021; Zhang et al., 2025; Yang et al., 2017). WAKs are a unique group of RLKs, which are localized in the cell wall and identified as key cell wall integrity sensors (Yao et al., 2025). WAKs are involved in signal transduction between the extracellular matrix and the intracellular compartments (Kanneganti and Gupta, 2008; Decreux and Messiaen, 2005). These kinases have been demonstrated to play a crucial role in plant cell expansion and elongation as well as in mediating plant defense responses to both abiotic stresses and fungal diseases (Lally et al., 2001; Wang et al., 2019b; Zuo et al., 2015).

In addition, WAKs possess Ser/Thr kinase activity and typically contain the extracellular WAK galacturonan-binding (GUB_WAK_bind), epidermal growth factor-like (EGF-like), transmembrane, and Pkinase domains (He et al., 1999). WAKs span the plasma membrane and extend into the extracellular region to bind tightly to the cell wall, providing a physical and signaling continuum between the cell wall and the cytoplasm (He et al., 1999). In *Arabidopsis*, *AtWAKs* were expressed at organ junctions, in shoot and root apical meristems, and in developing leaves. Additionally, their expression was induced in response to perturbations in the cell wall. The antisense of *AtWAKs* resulted in reduced WAK levels and lead to a loss of cell expansion (Wagner and Kohorn, 2001). In rice, *OsWAK12* was predominantly expressed in the roots. It positively regulated the growth and development of the rice root system by participating in auxin (IAA) signaling and modulating the expression of plasma membrane H^+^-ATPase-encoding genes, thereby influencing agronomic traits, including plant height and effective tillering (Hu, 2023). In cotton, 97% of *GhWAKs* were highly expressed in cotton fibers and ovules. Among them, 14 and 10 *GhWAKs* were found to possess putative gibberellin (GA) and IAA response elements in their promoter regions, respectively, and 13 and nine of these were significantly induced by GA and IAA treatment, respectively (Dou et al., 2021).

The *WAK* gene family plays a critical role in abiotic stress response. Previously, *OsWAK112d* had been identified as the candidate gene contributing to cold stress tolerance at the seedling stage of rice. Its expression is 200-fold higher in cold-tolerant plants than in cold-sensitive plants (Zhang et al., 2012). Furthermore, *Arabidopsis thaliana WALL-ASSOCIATED KINASE LIKE10* (*AtWAKL10*) has been shown to positively regulate salt stress tolerance in *Arabidopsis*, as evidenced by a reduced germination rate in the loss-of-function mutant *atwakl10* compared to the wild type under salt stress (Bot et al., 2019). In cotton, *GhWAKL26* was significantly activated by salt stress, with the transgenic plants displaying significantly increased dry weight, fresh weight, and root length. Further analysis showed that *GhWAKL26* primarily improves salt stress tolerance in cotton seedlings by regulating the Na^+^/K^+^ balance (Gao et al., 2024). Another study reported that *GhWAK9*, *GhWAK12*, *GhWAKL46*, and *GhWAKL47* were markedly upregulated under drought stress and that the corresponding proteins primarily localized to the plasma membrane (Zhang et al., 2021). Under heavy metal stress, the *Arabidopsis* protein AtWAK1 was found to accumulate in the plasma membrane region, and its overexpression restored aluminum (Al)-stress-mediated root growth inhibition (Sivaguru et al., 2003). Rice *OsWAK124* had been shown to localize to the cell wall, with overexpression lines exhibiting enhanced tolerance to heavy metals Al^3+^, Cu^2+^, and Cd^2+^ (Yin and Hou, 2017).

The *WAK* gene family also plays essential roles in biotic stress responses. In rice, *OsWAK91* is located within a major sheath blight disease (SB)-resistant quantitative trait locus (QTL) region on chromosome 9. Its resistant allele resulted in the loss of a stop codon, conferring resistance to SB in rice (Al-Bader et al., 2019). In maize, *ZmWAK* has been reported to activate *ZmSnRK1* after infection by the pathogen *Sporisorium reilianum* (Zhang et al., 2024). *ZmSnRK1* participates in gene transcription, resource allocation, energy metabolism, and programmed cell death (PCD), preventing pathogens from penetrating into the shoot apical meristem (SAM), thereby triggering smut disease resistance in plants (Zhang, 2016). In wheat, at least 30 *TaWAKs* were involved in the responses to *Fusarium* infection, with most of these genes contributing to pectin- and chitin-induced defense pathways. Furthermore, 45 *TaWAKs* has been detected within the resistance QTL regions of Fusarium head blight (FHB) disease (Xia et al., 2022).

With the advancement and extensive application of genome sequencing technologies, a substantial volume of plant genomic sequencing data has been released. Accordingly, genome-wide identification of gene families has become a crucial approach for functional gene discovery in plants (Bai et al., 2024; Wang et al., 2024a; Sun et al., 2023; Wang et al., 2024b). In previous studies, the functions of some maize *WAK/WAKL* genes have been investigated. For example, *ZmWAKL38*, *ZmWAKL42*, and *ZmWAKL52* were highly expressed in maize kernels (Hu et al., 2023). *ZmWAK02*, *ZMWAK17*, and *ZmWAK-RLK1* mediated resistance to maize gray leaf spot, stalk rot, and northern corn leaf blight, respectively (Dai et al., 2024; Zuo et al., 2022; Hurni et al., 2015). However, there is still a lack of a thorough examination of the physicochemical characteristics, evolutionary relationships, and transcriptome landscapes of the maize *WAK* gene family. In this study, the maize *WAK* gene family was systematically identified based on the maize B73_V5 genome, and extensive transcriptome data were utilized to explore the expression patterns of these genes in various tissues and under different stress conditions. Our findings might prove valuable for further elucidating the functional roles of maize *WAKs*.

## 2 Materials and methods

### 2.1 Identification of the *WAK* gene family in maize

The maize genome sequence (Zm-B73-REFERENCE-NAM-5.0) was downloaded from Ensembl Plants (https://plants.ensembl.org/index.html). The hidden Markov model (HMM) files of the *WAK* gene family were obtained from the InterPro database (https://www.ebi.ac.uk/interpro/). They included the typical domains GUB_WAK_bind (PF13947), EGF (PF07645), and Pkinase (PF00069). The typical domains were used as query files to identify the *WAKs* in the maize genome using the Advanced HMMER search tool in the TBtools-II software. The screening threshold value (E) was set to e^−3^. Subsequently, secondary identification of the *WAK* gene family was conducted using the InterPro (https://www.ebi.ac.uk/interpro/), SMART (http://smart.embl-heidelberg.de/), and NCBI CDD (https://www.ncbi.nlm.nih.gov/cdd) databases. Finally, 56 *WAK* genes were confirmed in the maize genome. The lengths and locations of these genes were obtained from the genome annotation files, and gene distribution on chromosomes was visualized using the MapChart2.32 software.

### 2.2 Physicochemical characteristics and phylogenetic analysis of the *WAK* gene family in maize

The physicochemical characteristics of the maize *WAK* gene family were assessed using the protein parameter Calc tool in the TBtools-II software. These characteristics included molecular weight (MW), isoelectric point (pI), instability coefficient, aliphatic index, and grand average of hydropathy (GRAVY). The subcellular localization of maize WAKs was predicted via the DeepLoc 2.1 website (https://services.healthtech.dtu.dk/services/DeepLoc-2.1/) and was visualized by the HeatMap tool in the TBtools-II software (Chen et al., 2023).

The *Arabidopsis* WAK protein sequence was obtained from the TAIR database (https://www.arabidopsis.org/). A multiple sequence alignment of the WAKs for maize and *Arabidopsis* was performed using the MUSCLE software. A phylogenetic tree was developed via the MEGA 11 software using the following parameters: Neighbor-joining (NJ) method, Poisson model, complete deletion, and 1000 bootstrap replications. The phylogenetic tree was constructed in the FigTree v1.4.4 software and optimized in EvolView v2 (He et al., 2016).

### 2.3 Gene structure and conserved motif analysis of the *WAK* gene family in maize

The structural analysis of the maize *WAK* gene family members was conducted using the GXF Stat tool in TBtools-II. The conserved motif analysis was performed using the Simple MEME Wrapper tool in TBtools-II. The parameters were set as follows: The maximum number of motifs was 10, the motif length was 6–50 amino acids, and the maximum value of E was 1×e^−10^. Finally, the phylogenetic tree, gene structure, and conserved motifs for the maize *WAK* gene family were visualized in the Gene Structure View tool of TBtools-II (Chen et al., 2023).

### 2.4 Gene duplication and collinearity analysis of the *WAK* gene family in maize

With the One Step MCScanX tool of TBtools-II (Chen et al., 2023), the gene duplication events and collinearity of the *WAK* gene family were analyzed across maize and other species, including dicotyledonous plants (*Arabidopsis thaliana* and *Glycine max*), and monocotyledonous plants (*Sorghum bicolor* and *Oryza sativa*). The duplication and collinear gene pairs of the *WAK* gene family were identified in the genomes of maize and other species, and the nonsynonymous (Ka) and synonymous substitution rates (Ks) of the collinear gene pairs were calculated using the Simple Ka/Ks Calculator tool in TBtools-II. The occurrence time of the gene duplication events was estimated by the equation T = (Ks/2λ) × 10^−6^, where λ = 6.161029 × 10^−9^ (Lynch and Conery, 2000). The visualization was performed using the Advanced Circos and Multiple Synteny Plot tools in TBtools-II.

### 2.5 Functional analysis of the *WAK* gene family in maize

The background file was downloaded from TBtools-II to analyze the function of the maize *WAK* gene family members. The maize protein sequences were uploaded to the egg-NOG-mapper website (http://eggnog6.embl.de/#/app/emapper) to obtain the annotation information. By inputting the above files into the GO Enrichment tool of Tbtools-II, the Gene Ontology (GO) enrichment analysis of maize *WAKs* was conducted. The enrichment result was displayed in a bubble plot format, which was drawn on the Weishengxin website (https://www.bioinformatics.com.cn/).

### 2.6 Expression analysis of maize *WAKs* in various tissues and under abiotic and biotic stresses

The maize transcriptome data were downloaded from the NCBI SRA database (https://www.ncbi.nlm.nih.gov/sra/), encompassing the various tissues (root, leaf, and seed), abiotic stresses (temperature, salt, drought, and waterlogging), and biotic stresses (smut, gray leaf spot, beet armyworm, and aphid) (Stelpflug et al., 2016; Li et al., 2020; Yu et al., 2020; Schurack et al., 2021; Yu et al., 2018; Tzin et al., 2015). With the Convert SRA to Fastq Files tool in TBtools-II, the transcriptome sequencing files were converted from the SRA format to the FASTQ format. The gene expression levels were calculated by aligning the FASTQ sequences to the maize B73_V5 genome in the Kallisto Super GUI Wrapper tool of TBtools-II. The calculation parameters were set as follows: Bias correction, kmer size was 31, BootStrap was 0, threadNum was 2, FragLen was 200, and LengthSD was 30. Finally, the gene expression patterns were visualized using the HeatMap tool in TBtools-II. The heatmaps of gene expression and expression changes under stress treatment were drawn based on normalized expression values, which were respectively calculated using the formulas log_2_(TPM + 1) and log_2_(Fold Change).

### 2.7 Expression analysis of maize *WAKs* under various hormonal treatments

In order to investigate the effects of hormones on the maize *WAK* gene family, the maize seedlings were treated with either indole-3-acetic acid (IAA), abscisic acid (ABA), or gibberellic acid (GA_3_). Briefly, the seeds of *Zea mays* cv. B73 were sown in commercial soil in the greenhouse, with a temperature of 28°C and a photoperiod of 16-h light/8-h dark. After 14 days of cultivation, the maize seedlings entered the three-leaf stage. The IAA, ABA, and GA_3_ were dissolved in 0.1% Tween-20 to prepare 100 µmol/L solutions of each. The three-leaf seedlings were treated with either of these three solutions. The controls were treated with 0.1% Tween-20. The leaves of seedlings were collected at 0, 3, 6, and 12 hours post-treatment. Each treatment was performed in three replicates, with three plants per replicate. All samples were flash-frozen in liquid nitrogen and subsequently stored at −80°C for further analysis.

RNA sequencing (RNA-seq) analysis was performed by the Gene Denovo company in Guangzhou. The total RNA of samples was extracted using the Trizol RNA extraction kit (Invitrogen, Carlsbad, CA, USA). The RNA quality was evaluated with the Agilent 2100 Bioanalyzer (Agilent Technologies, Santa Clara, CA, USA). The transcripts were randomly fragmented, generating a large number of fragments, approximately 200 nucleotides in length. The fragments were converted to cDNA, and the cDNA library was constructed using the NEBNext Ultra RNA Library Prep kit for Illumina (NEB#7530, New England Biolabs, Ipswich, MA, USA). RNA-seq was performed using the Illumina Novaseq 6000 system. Quality control of the RNA-seq data was performed using the fastp software (Chen et al., 2018). The clean reads were rapidly aligned to the reference genome with HISAT (Kin et al., 2015). Finally, the RNA-Seq data was quantified in the RSEM software, and the gene expression levels were expressed as fragments per kilobase of transcript per million mapped reads (FPKM) (Li and Dewey, 2011). The expression patterns of *WAK* gene family members were visualized in Graphpad Prism 9.5, and the significance analysis of these genes was performed using SPSS 12.

### 2.8 Roles of maize *WAKs* under abiotic and biotic stresses, and hormonal treatments

We selected genes with high expression levels (TPM >10) and the most significant variation under different stresses for further analysis (**Supplementary Tables S11**-**S18**). A total of 34 genes were selected, including 6 genes under temperature stress, 9 genes under salt stress, 6 genes under drought stress, 11 genes under waterlogging stress, 16 genes under smut stress, 18 genes under gray leaf spot stress, 9 genes under beet armyworm stress, 12 genes under aphid stress, and 6 genes under hormonal treatments (IAA, ABA, GA). Except for *ZmWAK15*, *22*, *25*, *34*, and *48*, the remaining genes were significantly induced by multiple stresses.

To elucidate the functions of maize *WAK* genes in abiotic and biotic stresses as well as hormonal treatments, we generated a heatmap depicting the expression patterns of 34 genes. For each gene, the expression value used in the heatmap was determined by the most significant change [Log_2_(Fold Change)] observed under the respective stresses. For instance, under temperature stress [encompassing the normal temperature (25°C), the low temperature (16°C), the extremely low temperature (4°C), the moderately low temperature (10°C), the high temperature (37°C), the moderately high temperature (42°C), the extremely high temperature (48°C)], the heatmap value for each gene was derived from the condition exhibiting the maximum expression variation. Finally, the gene expression patterns were visualized using the HeatMap tool in TBtools-II.

### 2.9 Construction of the maize WAK protein interaction network

To investigate the interaction among the maize *WAK* gene family members, the corresponding protein sequences were uploaded to the Orthovenn2 database (https://orthovenn2.bioinfotoolkits.net/home), and were aligned with the protein sequences of *A. thaliana* and rice for homology analysis (Wang et al., 2019a).

The maize WAK protein interaction analysis was conducted in the STRING website (https://cn.string-db.org/), with a credibility value of 0.4, and the number of interacting proteins was limited to within 20. The maize WAK protein interaction network was constructed using the Cytoscape3.8.2 software (Shannon et al., 2003).

## 3 Results

### 3.1 Identification of the *WAK* gene family in the maize genome

**Figure 1:**
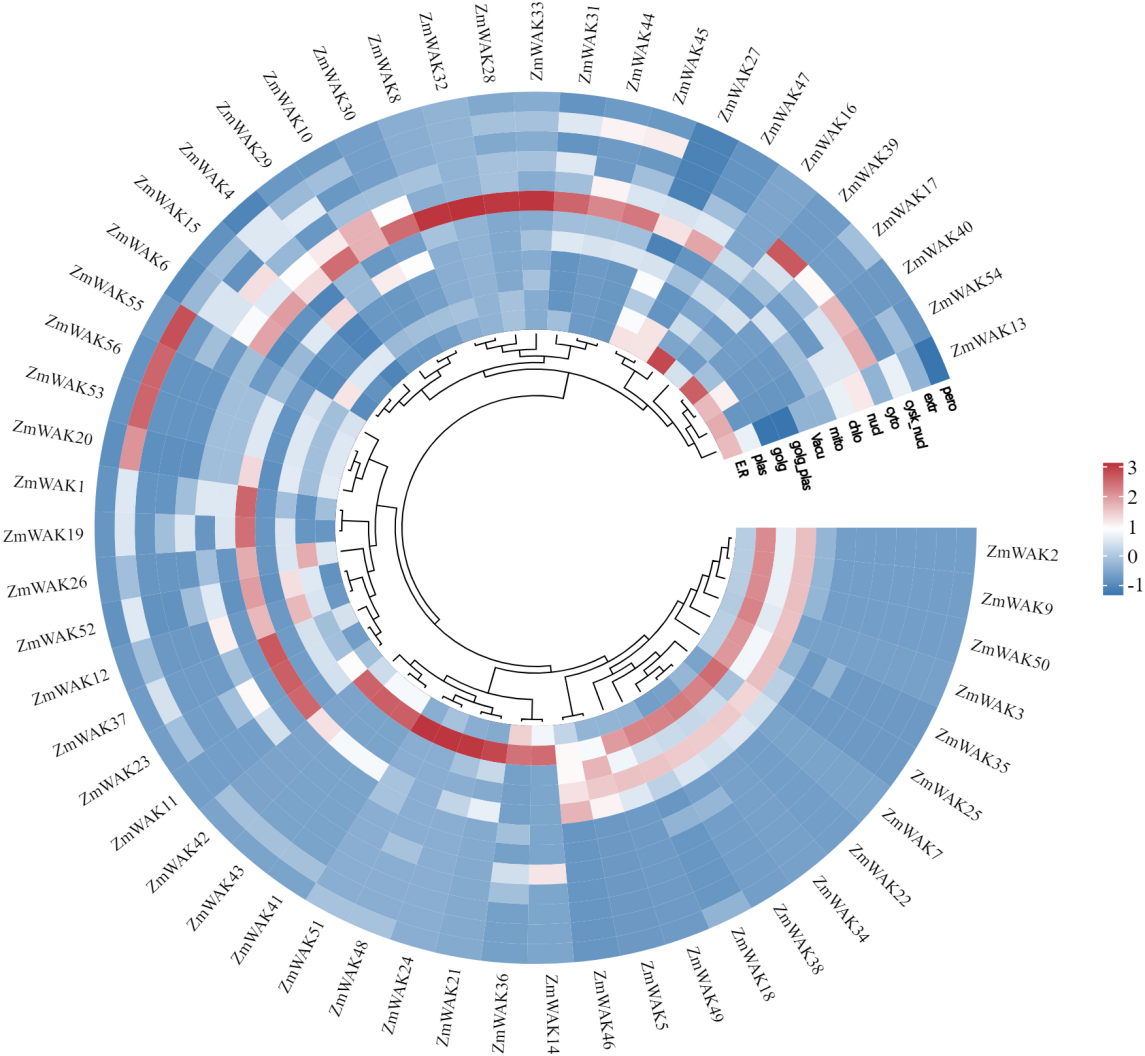
Heat map of the subcellular localization of *ZmWAK* genes in plants. E.R, Endoplasmic Reticulum; plas, plasma membrane; Golg, Golgi apparatus; Golg_plas, Golgi membrane; Vacu, vacuole; mito, mitochondria; chlo, chloroplast; nucl, nucleus; cyto, cytosol; cysk_nucl, nuclear cytoskeleton; extr, extracellular matrix; pero, peroxisome. The minimal functional presence of the gene is indicated by blue, and the maximum functional significance of the gene is represented by red.

A total of 56 *WAK* genes were identified at the whole-genome level in maize. According to their locations on the chromosome, the genes were named *ZmWAK1*–*ZmWAK56* (**Supplementary Table S1**). The polypeptides encoded by *ZmWAKs* contained 319–1016 amino acids, with the longest being ZmWAK29. The MW of the encoded proteins ranged from 35.20 kDa to 110.83 kDa. Furthermore, their theoretical pI ranged from 4.87 to 9.70, indicating a predominantly acidic nature. The instability index varied from 32.11 to 60.50, with 46.43% for unstable protein. The aliphatic index ranged between 69.85 and 94.22. Finally, the GRAVY values ranged from -0.504 to 0.061, with negative values for 91.07% WAKs, reflecting their hydrophilic nature. The subcellular localization predictions indicated that maize WAK proteins predominantly localized to the plasma membrane, chloroplast, and vacuole, with lesser function in the endoplasmic reticulum, cytosol, extracellular matrix, and nucleus (**Figure 1**).

### 3.2 Chromosomal location of *ZmWAKs*

**Figure 2:**
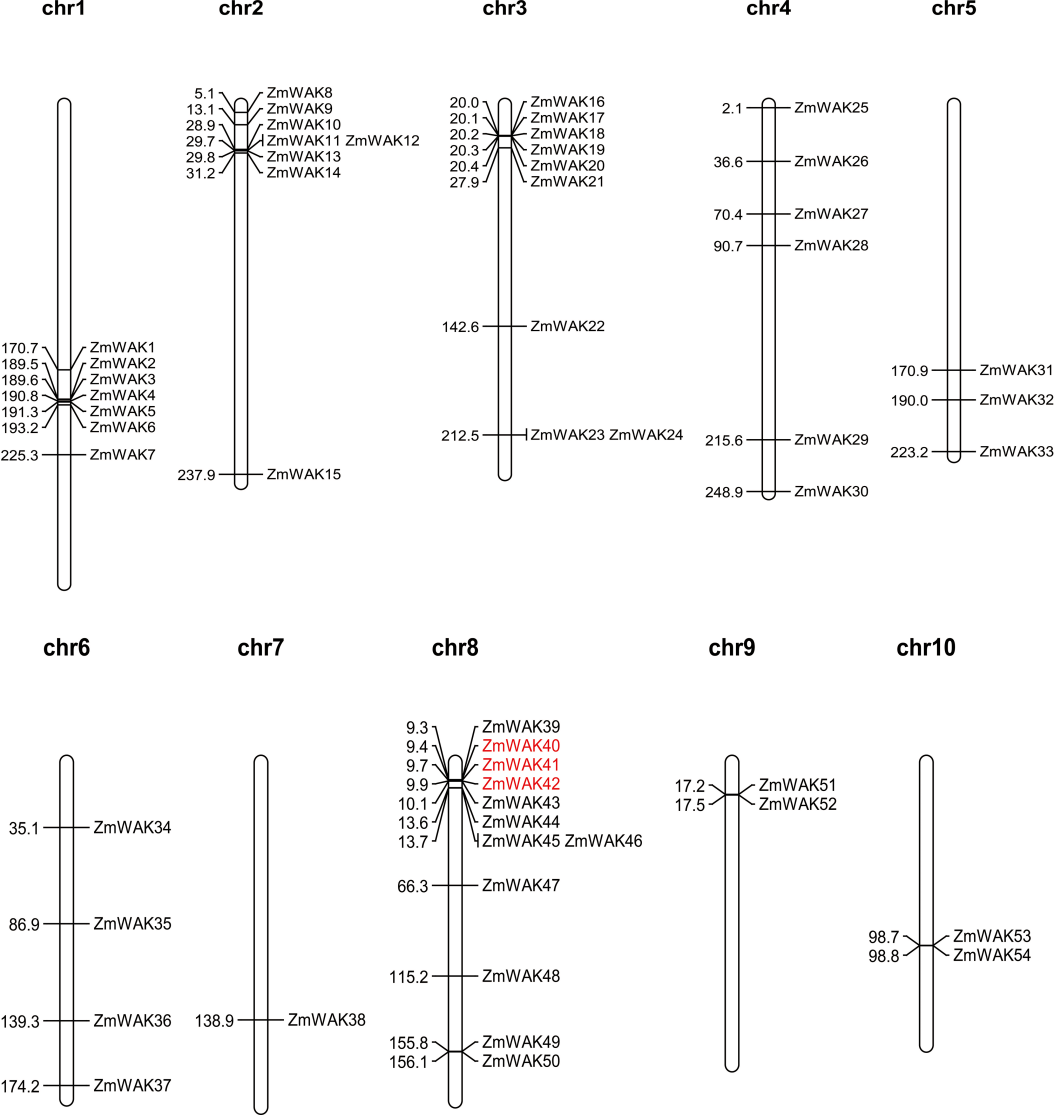
Distribution of *ZmWAKs* in maize genome. Red color represents these genes underwent tandem duplication.

The chromosomal location of maize *WAK* genes (Zm-B73-REFERENCE-NAM-5.0) was analyzed to understand the distribution of the maize *WAK* gene family across chromosomes (**Supplementary Table S1**, **Figure 2**). Among the 56 identified genes, 54 *ZmWAKs* were unevenly distributed on all maize chromosomes. Chromosome 8 contained the highest number of *ZmWAKs* (*n* = 12), followed by chromosomes 3, 2, 1, 4, 6, and 5 (*n* = 9, 8, 7, 6, 4, and 3). Chromosomes 7, 9, and 10 possessed the fewest *ZmWAKs* (*n* = 1 or 2). The WAKL proteins share similar structural features with the WAK proteins. In a previous study, a total of 58 *ZmWAKL* genes were identified in the maize genome of the Zm-B73-REFERENCE-GRAMENE-4.0 version, and were designated as *ZmWAKL1* to *ZmWAKL58* (Hu et al., 2023). The 58 *ZmWAKL* genes are unevenly distributed across the 10 chromosomes, with the highest number (15 genes) located on chromosome 8 and the lowest number (1 gene) on chromosome 7. The variation in the number of *ZmWAK/WAKL* genes may be due to the use of different genome versions during the identification process of the gene family.

To obtain the evolutionary relationships among the *ZmWAKs*, we detected gene duplication events, including both segmental (SD) and tandem duplication (**Table 1**). Nine pairs of SD genes, including *ZmWAK6/38*, *ZmWAK12/32*, *ZmWAK16/45*, *ZmWAK23/37*, *ZmWAK23/47*, *ZmWAK23/49*, *ZmWAK37/47*, *ZmWAK49/37*, and *ZmWAK49/47*, and two pairs of TD genes, including *ZmWAK40/41* and *ZmWAK41/42* were identified in maize. *ZmWAK40/41* and *ZmWAK41/42* are two connected tandem duplication events, which together make these genes tandem triplicates. *ZmWAK23*, *ZmWAK37*, *ZmWAK47*, and *ZmWAK49* exhibited 2–3 SD events, indicating these genes were the most active in the expansion of the maize WAK gene family. The SD and TD events occurred over 14.77–226.02 million and 2.55–2.76 million years ago, respectively. The Ka and Ks values of the 11 duplication gene pairs were calculated to estimate the selection pressure in gene differentiation. Four gene pairs exhibited Ka/Ks values greater than 1, suggesting that these gene pairs may have undergone positive selection. This indicates that the amino acid substitutions in these genes could potentially give rise to novel protein functions, thereby facilitating the adaptation of the organisms to environmental changes.

**Table 1:** Ka/Ks analysis of duplicated *ZmWAK* gene pairs.

Duplicated gene pairs	Ka	Ks	Ka/Ks	Duplicated type	Selection type	Time (Mya)
*ZmWAK40/ZmWAK41*	0.009	0.036	0.259	Tandem	purifying selection	2.766
*ZmWAK41/ZmWAK42*	0.013	0.033	0.403	Tandem	purifying selection	2.555
*ZmWAK6/ZmWAK38*	0.666	1.094	0.609	Segmental	purifying selection	84.138
*ZmWAK12/ZmWAK32*	0.845	0.946	0.894	Segmental	purifying selection	72.734
*ZmWAK16/ZmWAK45*	0.467	2.938	0.159	Segmental	purifying selection	226.018
*ZmWAK23/ZmWAK37*	0.976	1.061	0.920	Segmental	purifying selection	81.605
*ZmWAK23/ZmWAK47*	1.043	0.905	1.152	Segmental	positive selection	69.633
*ZmWAK23/ZmWAK49*	0.942	1.095	0.860	Segmental	purifying selection	84.260
*ZmWAK37/ZmWAK47*	0.250	0.192	1.302	Segmental	positive selection	14.773
*ZmWAK49/ZmWAK37*	0.578	0.261	2.218	Segmental	positive selection	20.060
*ZmWAK49/ZmWAK47*	0.447	0.218	2.053	Segmental	positive selection	16.752

Ka and Ks indicate the nonsynonymous and synonymous which are used to determine the selective pressure after duplication. Ka/Ks = 1 indicates the neutral selection, Ka/Ks > 1 indicates the positive selection, Ka/Ks < 1 indicates the purifying selection. The duplication date (Million years ago, Mya) is calculated by the formula: T = (Ks/2λ)×10^-6^, where λ = 6.5×10^-9^.

### 3.3 Phylogenetic analysis of the maize *WAK* gene family

**Figure 3:**
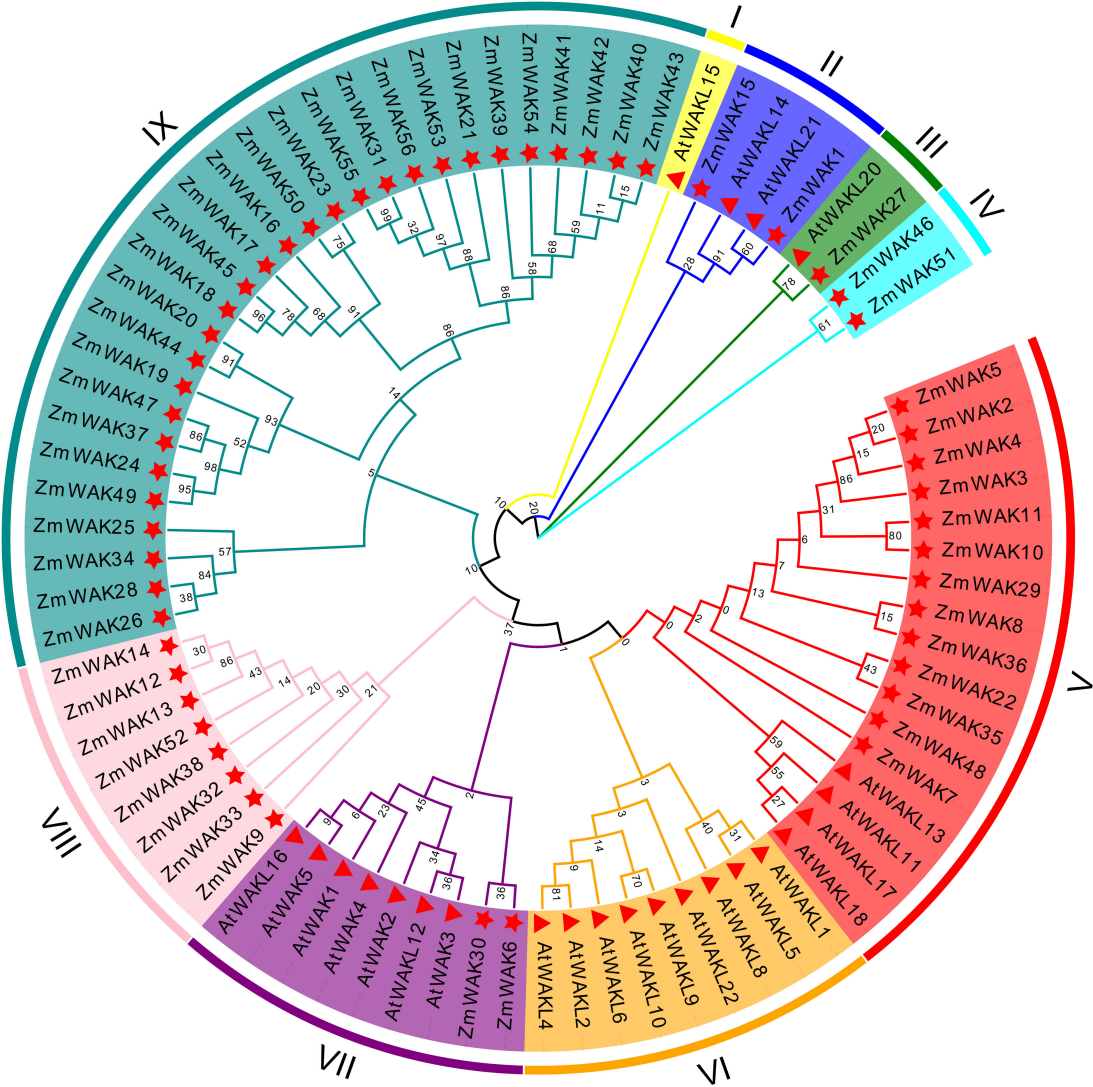
Phylogenetic relationships of the WAK proteins from *Arabidopsis* (At) and maize (Zm). Distinct color blocks represent different subgroups. The Star shape represents maize WAK proteins, and the triangle represents *Arabidopsis* WAK proteins.

In order to elucidate the phylogenetic relationships of *ZmWAKs*, a phylogenetic analysis was conducted with the *WAK* gene family members from *Arabidopsis* and maize. A total of nine subgroups were identified: I, II, III, IV, V, VI, VII, VIII, and IX (**Figure 3**). Among them, subgroup IX was the largest, comprising 28 *ZmWAKs*, followed by subgroup V, containing 13 *ZmWAKs* and four *AtWAKs/WAKLs*; subgroup VII, containing two *ZmWAKs* and seven *AtWAKs/WAKLs*; subgroup VI, containing nine *AtWAKs/WAKLs*; subgroup VIII, containing eight *ZmWAKs*; subgroup II, containing two *ZmWAKs* and two *AtWAKs/WAKLs*; subgroup IV, containing two *ZmWAKs*; subgroup III, containing one *ZmWAK* and one *AtWAK/WAKL*; and subgroup I, containing one *AtWAK/WAKL*. These data indicate that the expansion of the plant *WAK* gene family occurred unevenly across different species during evolution. In addition, subcellular localization reveals that 22 ZmWAK proteins are localized to the plasma membrane, predominantly in subgroups IV, V, VIII, and IX, which account for 50.00%, 53.84%, 37.50%, and 39.28% of the genes in these subgroups, respectively (**Supplementary Table S1**). In a previous study, 58 *ZmWAKL* genes in maize were identified three main clusters: Group I, Group II, and Group III, with 29, 19, and 10 genes, respectively (Hu et al., 2023). In this study, 56 *ZmWAK* genes were identified, of which 8, 13, and 28 were respectively assigned to subgroups V, VIII, and IX, with the remaining 7 genes allocated to other subgroups. This difference in grouping may result from the use of both *Arabidopsis* and maize *WAK/WAKL* genes in this study.

### 3.4 Gene structure and conserved motif analysis of the maize *WAK* gene family

To further explore the evolution of the *ZmWAK* gene family, the gene structure and conserved motifs of *ZmWAKs* were analyzed. As shown in **Supplementary Table S1**, a total of five typical domains were detected in *ZmWAKs*, including signal peptide, GUB_WAK_bind domain, EGF domain, Pkinase_Tyr domain, and transmembrane domain. We observe that 28.57%, 21.42%, 17.85%, and 32.14% of *ZmWAKs* contain all five, four, three, and two typical domains, respectively. No ZmWAKL proteins were identified, because all identified ZmWAK proteins contain at least one extracellular domain (GUB_WAK_bind, EGF) and an intracellular domain (Pkinase_Tyr), based on the identification method used by previous researchers (Yang et al., 2021; Li et al., 2025). However, in a previous study, 58 ZmWAKL proteins were identified, which contained EGFs and a protein kinase domain (Hu et al., 2023). In this study, all 56 ZmWAK proteins contain an extracellular domain (GUB_WAK_bind,EGF) and an intracellular domain (Pkinase_Tyr). This suggests that the differences in gene number between the two studies may be attributed to variations in identification methods and screening criteria.

**Figure 4:**
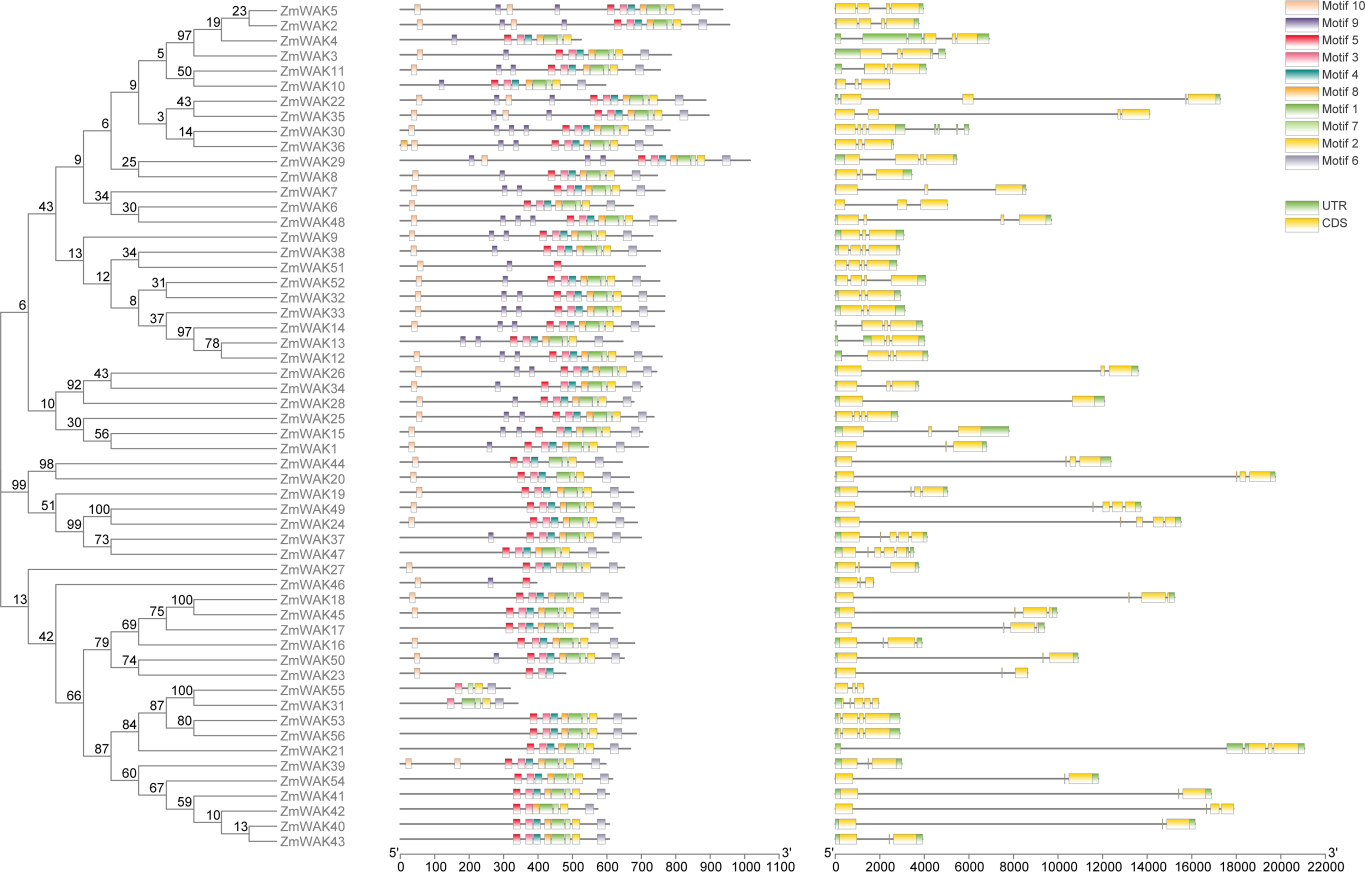
The gene structure and conserved motifs of *ZmWAK* gene family. From left to right, they are as follows: the phylogenetic tree of the 56 ZmWAK proteins, the motif distribution of the 56 ZmWAK proteins, The exon-intron structure of the *ZmWAK* genes.

The ZmWAK proteins in the same group displayed similar conserved domains and structures. For example, groups V and IX are the two largest subgroups within the maize *WAK* gene family, comprising 13 and 28 ZmWAK proteins, respectively. In subgroup V, 76.92% of the ZmWAK proteins harbor the signal peptide, GUB_WAK_bind, EGF, transmembrane, and Pkinase_Tyr domains, with either two GUB_WAK_bind domains or two EGF domains present. In subgroup IX, 89.28% of the ZmWAK proteins are devoid of the EGF domain, while 42.8% of the ZmWAK proteins contain the WAK_assoc domain. Furthermore, we find that AtWAKL20 and ZmWAK27 (in subgroup III) and AtWAKL21 and ZmWAK1 (in subgroup II) are sub-clustered despite having different domain compositions. We speculate that these genes may have retained conserved domains or amino acid sequences during evolution. Conserved motif analysis reveals that around 89.29% of *ZmWAKs* contain three or four exons, whereas 10.71% of *ZmWAKs* contain two or five exons (**Supplementary Table S1**, **Figure 4**). In a previous study, the number of exons in *ZmWAKL* genes varied from 2 to 5 (Hu et al., 2023). This is similar to the results of this study. Additionally, Similar intron-exon distribution has been reported in other plants, implying that this feature of the *WAK* gene family might be conserved across different plant species.

A total of ten conserved motifs were predicted via the MEME analysis (**Supplementary Table S2**). Most of the *ZmWAKs* from subgroups II, V, and VIII contained all ten motifs. Around 78.57% and 46.42% of *ZmWAKs* from subgroup IX lacked motifs 9 and 10, respectively. *ZmWAKs* from subgroup IV contained the least number of motifs, harboring only motifs 5, 9, and 10. *ZmWAKs* from subgroups III and VII displayed a trend of losing motifs or having multiple copies of motif 9 (**Supplementary Tables S2**, **S3**, **Figure 4**). The gene structure and motif composition of ZmWAKs were similar within the same subgroup, indicating functional redundancy in plant growth and development. In a previous study, there were ten motifs identified in ZmWAKL proteins that are associated with the wall-associated kinase or protein kinase domain (Hu et al., 2023). A similar distribution of conserved motifs was observed between the *ZmWAKL* and *ZmWAK* gene families.

### 3.5 Analysis of the *cis*-acting elements of the maize *WAK* gene family

**Figure 5:**
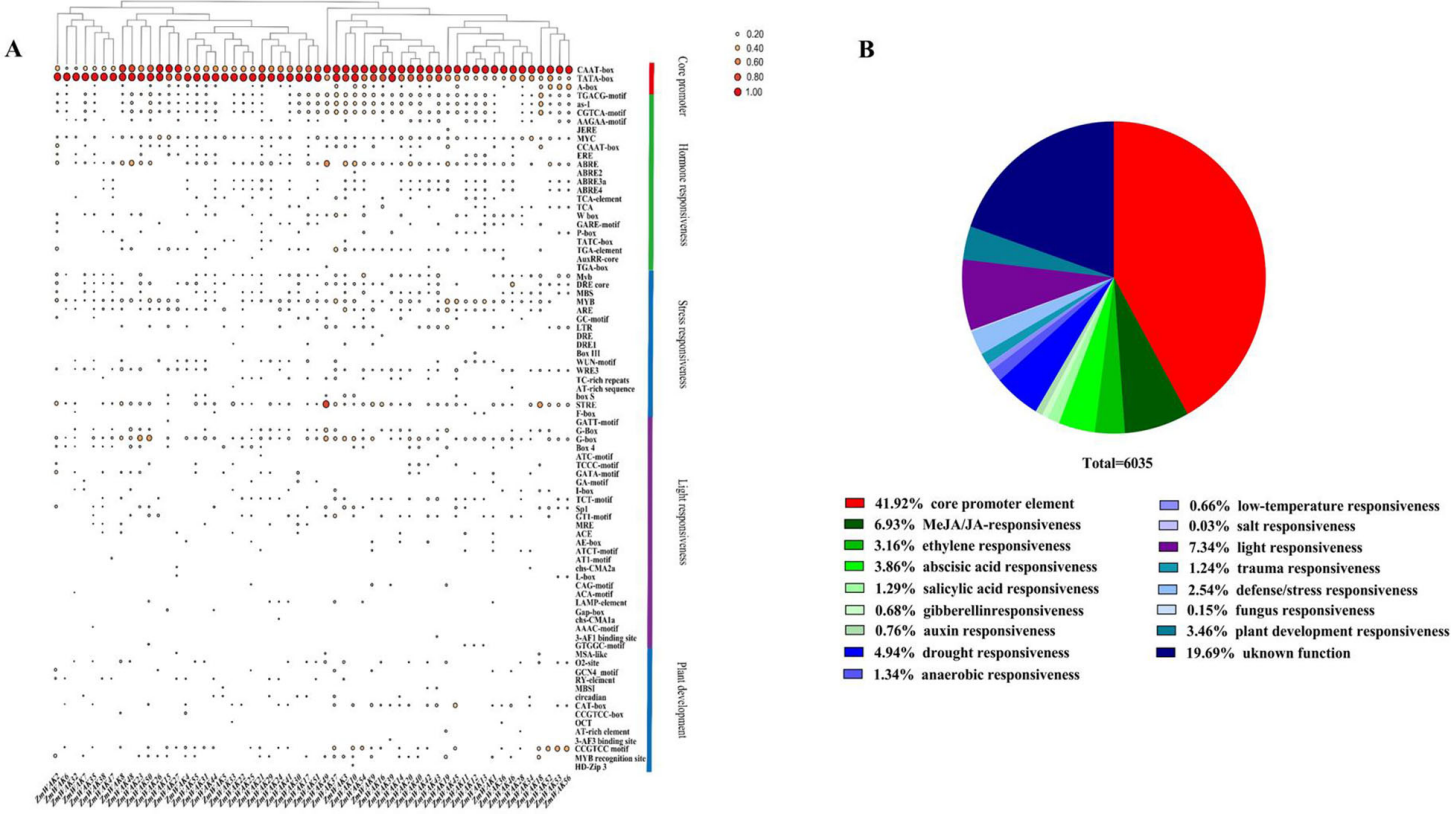
Predicted *cis*-elements in promoter regions of *ZmWAK* genes. **(A)** Distribution of different *cis*-regulatory elements. **(B)** The relative proportion of various *cis*-elements within promoter regions.

A total of 107 distinct types of *cis*-acting elements were identified in the promoter regions of maize *WAK* genes, including 82 elements with known functions and 25 elements with unknown functions (**Supplementary Tables S4**, **S5**, **Figure 5A**). A significant proportion (41.92%) of *cis*-acting elements were core promoter elements, followed by light-responsive elements (7.34%). Furthermore, specific *cis*-acting elements were predicted to participate in the responses to various hormones (methyl jasmonate/jasmonic acid (MeJA/JA), ABA, ethylene (ETH), salicylic acid (SA), IAA, and GA) and stress factors (drought, anaerobic, injury, low temperature (LT), salt, and fungus), defense responses, and plant development processes. Among them, the defense-responsive elements, elements related to plant development, and abiotic stress-responsive elements (MeJA/JA, ABA, ETH, and drought) were widely distributed in the promoter sequences of *ZmWAKs* (found in 2.54%, 3.46%, 6.93%, 3.86%, 3.16%, and 4.94% of the sequences, respectively; **Figure 5B**). This result suggests that *ZmWAKs* might be involved in stress responses and might regulate plant growth and development via hormonal pathways.

### 3.6 GO annotation analysis of the maize *WAK* gene family

Upon GO enrichment analysis, a total of eight *ZmWAK* genes were successfully annotated (**Supplementary Figure S1**). The molecular function enrichment analysis revealed that the genes were primarily associated with guanylate cyclase, phosphorus-oxygen lyase, and cyclase activities. The cellular component enrichment analysis showed that they were mainly distributed in the plant cell wall, plasma membrane, and cell periphery. The biological process enrichment analysis highlighted a significant enrichment of the genes in cGMP biosynthesis, cyclic purine nucleotide metabolism, and cyclic nucleotide metabolism/biosynthesis.

### 3.7 Collinearity analysis of the maize *WAK* gene family

**Figure 6:**
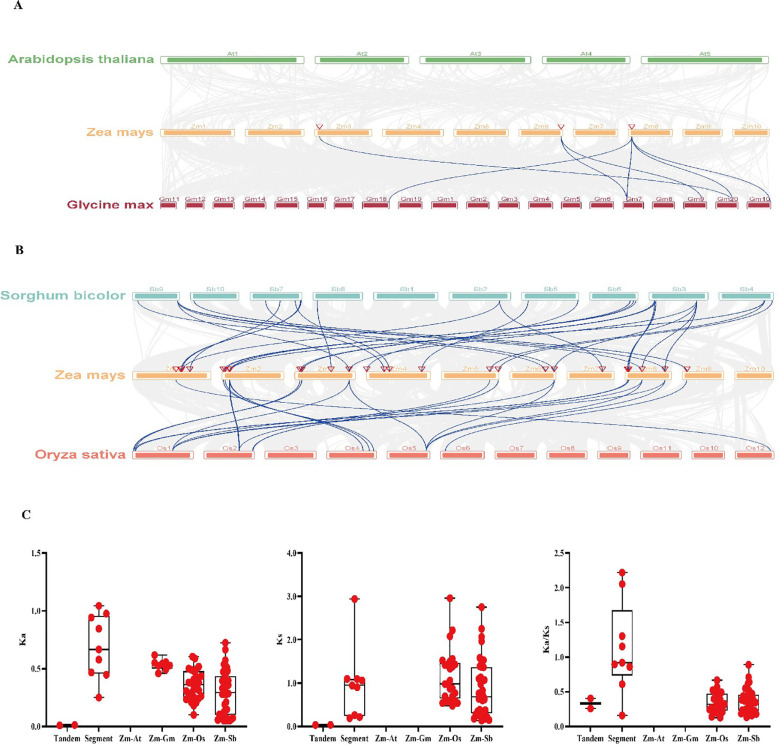
Gene duplication and collinearity of the *WAK* genes in maize. **(A)** The collinearity analysis of *WAK* genes among maize, *Arabidopsis* and soybean. **(B)** The collinearity analysis of *WAK* genes among maize, rice and sorghum. **(C)** The tandem repeats, segment repeats, and repeats among maize, *Arabidopsis* (Zm-At), soybean (Zm-Gm), rice (Zm-Os) and sorghum (Zm-Sb), respectively.

To understand the collinearity of *ZmWAKs* in different species, a collinearity analysis was performed between maize *WAKs* and corresponding genes of monocotyledonous and dicotyledonous plants. As shown in **Figure 6**, a total of 76 interspecific collinear relationships were identified with maize *WAKs*, including eight, 27, and 41 collinear links with soybean, rice, and sorghum, respectively (**Supplementary Table S6**). However, no collinear links were detected between maize and *Arabidopsis*. Further analysis found that *ZmWAK37* exhibited the highest collinearity, with six collinear links, followed by *ZmWAK11*, *ZmWAK14*, *ZmWAK44*, *ZmWAK47*, and *ZmWAK49* (**Supplementary Table S6**). The Ka/Ks analysis revealed higher Ka, Ks, and Ka/Ks values for SDs than TDs for maize *WAKs*. The average Ks value of the maize gene pairs with rice homologs was markedly greater than 1. However, the Ka, Ks, and Ka/Ks values of the maize gene pairs with soybean and sorghum homologs were <1. In addition, based on these data, it was calculated that maize diverged from rice and sorghum around 227.56 and 211.68 million years ago, respectively.

### 3.8 Tissue-specific expression analysis of *ZmWAKs*

#### 3.8.1 Expression of *ZmWAKs* in the root

**Figure 7:**
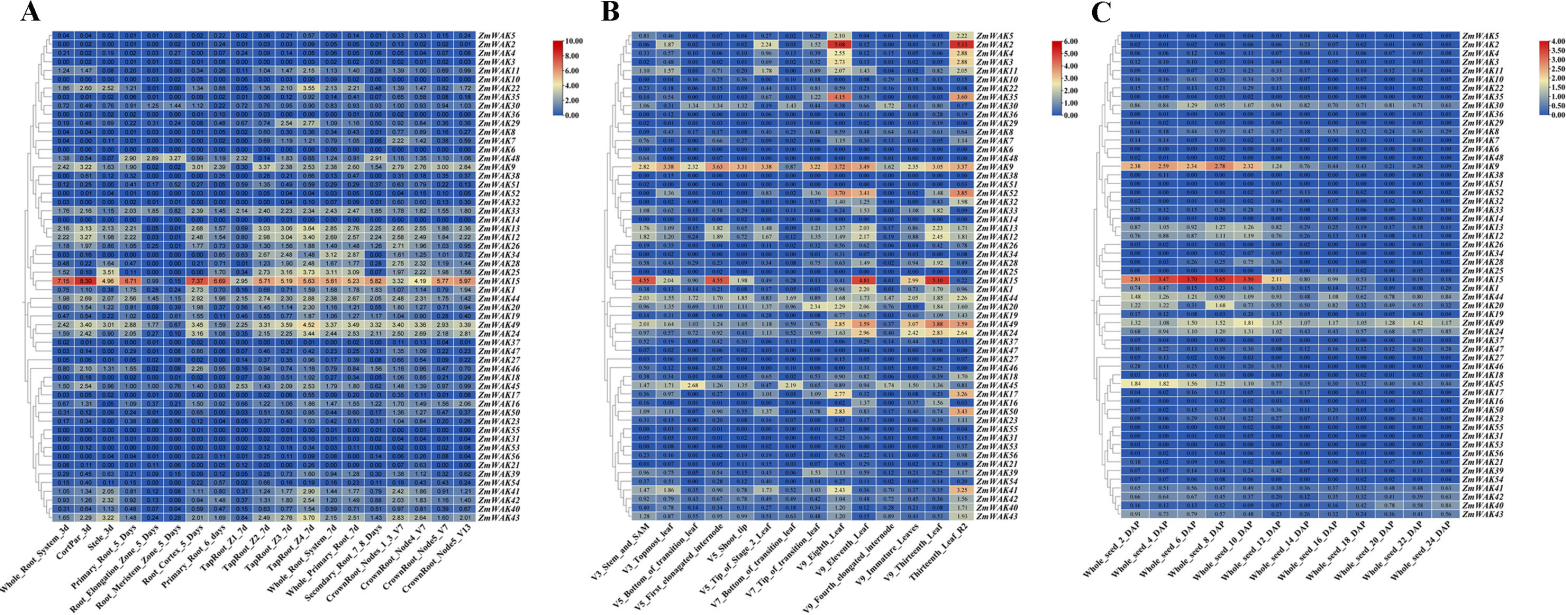
Maize *WAK* gene family expression heat map in maize development. **(A)**Maize *WAK* gene family expression heat map in root development. **(B)** Maize *WAK* gene family expression heat map in leaf development. **(C)** Maize *WAK* gene family expression heat map in seed development. The numbers in the colored boxes are calculated based on the transcript per million reads (TPM) values. The calculation formula is log_2_ (TPM + 1).

The expression of *ZmWAKs* in the root was analyzed using the transcriptome data (PRJNA171684) derived from a previous study (Stelpflug et al., 2016). The expression levels of 34 *ZmWAKs* identified in the root exceeded 1 transcript per million reads (TPM > 1) (**Supplementary Table S7**; **Figure 7A**). Among them, the highest expression levels were observed for *ZmWAK15* (TPM > 314) and several other genes: *ZmWAK13*, *ZmWAK22*, *ZmWAK25*, *ZmWAK43*, *ZmWAK49* (TPM > 10). At 5 days post-sowing, *ZmWAK15* was highly expressed in various parts of the root, except the root elongation and meristem zone. At 7 days post-sowing, *ZmWAK13*, *ZmWAK22*, *ZmWAK25*, *ZmWAK43*, and *ZmWAK49* displayed peak expression levels at the Z4 region of taproot. However, these five genes were hardly expressed in the root elongation and meristem zone at 5 days post-sowing.

#### 3.8.2 Expression of *ZmWAKs* in the leaf

The expression of *ZmWAKs* in the leaf was analyzed using previously published transcriptome data (Stelpflug et al., 2016). The expression levels of 32 *ZmWAKs* identified in the leaf were >1 (**Supplementary Table S8**; **Figure 7B**). Among them, the highest expressions were observed for *ZmWAK2*, *ZmWAK9*, *ZmWAK15*, *ZmWAK35*, *ZmWAK49*, and *ZmWAK52* (TPM > 10). *ZmWAK15* expression peaked in V3 stem and SAM, V5 first elongated internode, and V9 eleventh and thirteenth leaves. *ZmWAK2*, *ZmWAK35*, and *ZmWAK52* were highly expressed in V9 eighth leaf and R2 thirteenth leaf. *ZmWAK9* and *ZmWAK49* displayed peak expression levels in V9 eighth leaf and thirteenth leaf, respectively.

#### 3.8.3 Expression of *ZmWAKs* in the seed

Using previously published transcriptome data, ten *ZmWAKs* were found to be highly expressed in the seed (TPM > 1) (Stelpflug et al., 2016). Among them, *ZmWAK9* and *ZmWAK15* exhibited the highest expression levels in the early stages of seed growth, such as 2-, 4-, 6-, 8-, and 10 days post-pollination (**Supplementary Table S9**; **Figure 7C**).

### 3.9 Expression patterns of *ZmWAKs* under abiotic stresses

#### 3.9.1 Expression patterns of *ZmWAKs* under temperature stress

**Figure 8:**
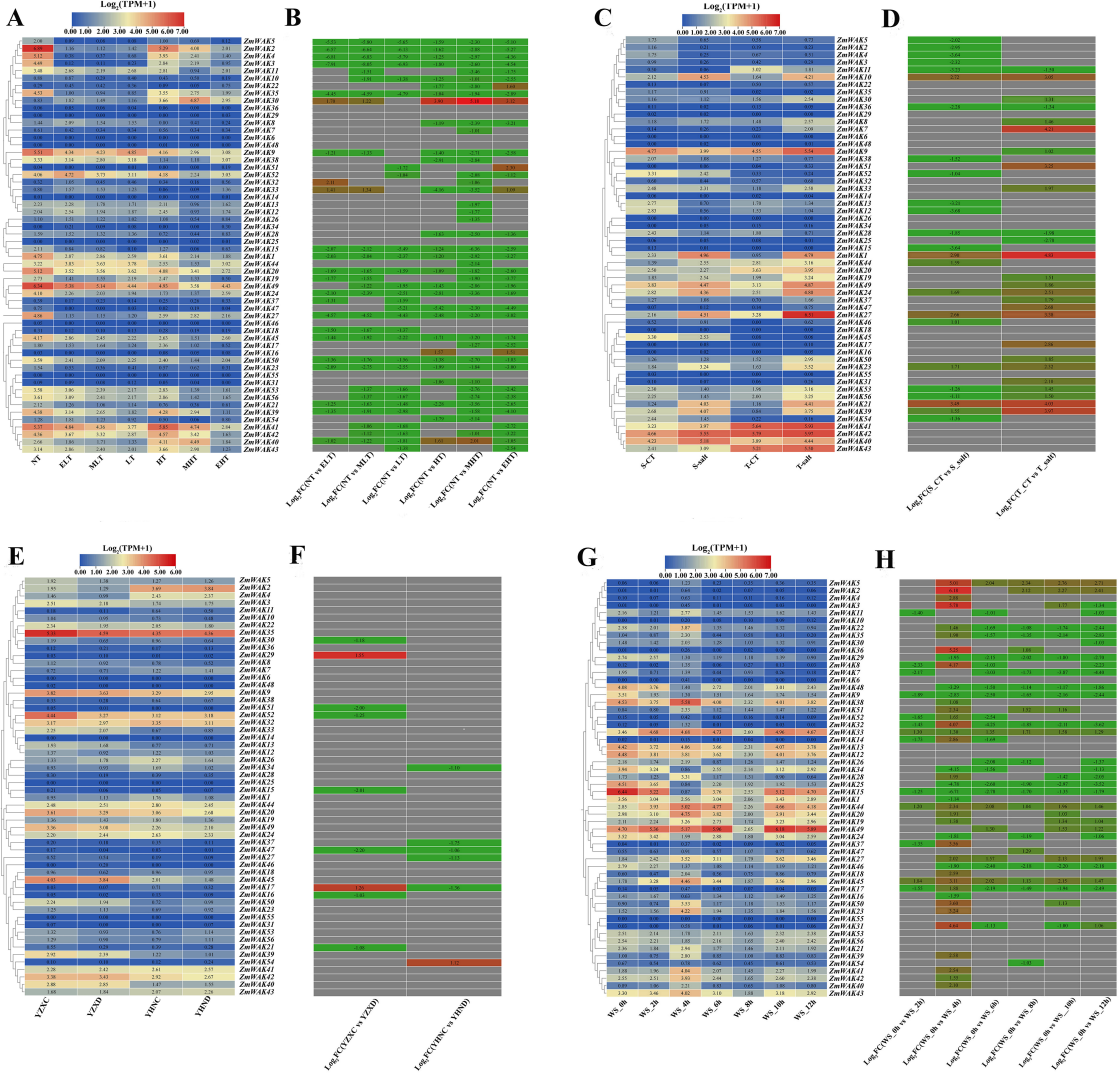
Maize *WAK* gene family expression heat map in abiotic stress. **(A)** Temperature stress expression pattern of WAK genes in maize. NT represents the normal temperature (25 °C), LT represents the low temperature (16 °C), ELT represents the extremely low temperature (4 °C), MLT represents the moderately low temperature (10 °C), HT represents the high temperature (37 °C), MHT represents the moderately high temperature (42 °C), EHT represents the extremely high temperature (48 °C). **(B)** Expression fold changes of the maize *WAK* gene family in temperature stress. **(C)** Salt stress expression pattern of *WAK* genes in maize. T-salt represents the inbred salt-tolerant maize line L87 treated with salt (220 mM NaCl); S-salt represents the salt sensitivity inbred line L29 treated with salt (220 mM NaCl), T−CT represents the inbred salt-tolerant maize line (L87) treated with water, S−CT represents the inbred salt-sensitive maize line (L29) treated with water. **(D)** Expression fold changes of the maize *WAK* gene family in salt stress. **(E)** Drought stress expression pattern of *WAK* genes in maize. YHND represents the drought-tolerant hybrid (ND476) treated with drought, YHNC represents the drought-tolerant hybrid (ND476) treated with water, YZXD represents the drought-intolerant hybrid (ZX978) treated with drought, YZXC represents the drought-intolerant hybrid (ZX978) treated with water. **(F)** Expression fold changes of the maize *WAK* gene family in drought stress. **(G)** Waterlogging stress expression pattern of *WAK* genes in maize. WS_0h represents waterlogging 0 hour, WS_2h represents waterlogging 2 hours, WS_4h represents waterlogging 4 hours, WS_6h represents waterlogging 6 hours, WS_8h represents waterlogging 8 hours, WS_10h represents waterlogging 10 hours, WS_12h represents waterlogging 12 hours. **(H)** Expression fold changes of the maize *WAK* gene family in Waterlogging stress. There are two figures in each stress treatment—the left figure represents the log_2_ (TPM + 1) and the right figure represents the log_2_ (Fold Change), indicated in red for up-regulation and green for down-regulation. When the absolute value of log_2_ (Fold Change) is greater than 1, it will be shown in the figure.

The changes in the expression of *ZmWAKs* in response to temperature stress were assessed using the transcriptome data (PRJNA645274) derived from a previous study (Li et al., 2020). A total of 38 *ZmWAKs* genes were expressed in maize seedling leaves under temperature stress (TPM > 1) (**Supplementary Table S10**; **Figures 8A, B**). Of them, 23 *ZmWAKs* displayed highly expression levels (TPM > 10). Among these 23 genes, compared to normal temperature (NT) condition, 16 genes were significantly downregulated, and one gene (*ZmWAK30*) was significantly upregulated under both low-and high-temperature conditions. The expression level of *ZmWAK40* was upregulated under high temperature (HT) and moderately high temperature (MHT) conditions compared to NT. *ZmWAK41*, *ZmWAK42* and *ZmWAK43* were upregulated under HT condition. *ZmWAK52* was upregulated under HT and extremely low temperature (ELT) conditions. *ZmWAK44* exhibited contrasting expression patterns under low-and high-temperature conditions: its expression level rose with decreasing temperature and fell with increasing temperature. Under temperature stress, eight *ZmWAKs* displayed the most significant changes. Among them, *ZmWAK1*, *ZmWAK2*, *ZmWAK3*, *ZmWAK4*, *ZmWAK24*, *ZmWAK27*, and *ZmWAK35* exhibited 51.47–99.62% downregulation compared to NT condition, while *ZmWAK30* exhibited 0.58–35.37-fold upregulation.

#### 3.9.2 Expression patterns of *ZmWAKs* under salt stress

The changes in the expression of *ZmWAKs* in response to salt stress were assessed using the transcriptome data (PRJNA414300) downloaded from the NCBI website (http://www.ncbi.nlm.nih.gov). A total of 36 *ZmWAKs* genes were expressed under salt stress (TPM > 1) (**Supplementary Table S11**; **Figures 8C, D**). Among them, 14 *ZmWAKs* demonstrated high expression levels, with TPM values exceeding 10. Nine *ZmWAKs* exhibited significant differential expression in maize inbred lines after salt treatment (|Log_2_ (Fold Change)| > 1). Among the differentially expressed genes, eight genes (*ZmWAK1*, *ZmWAK10*, *ZmWAK21*, *ZmWAK23*, *ZmWAK24*, *ZmWAK27*, *ZmWAK39*, and *ZmWAK49*) were significantly upregulated under salt stress. The levels of these genes increased by 0.59–10.23-fold in the salt-sensitive (S-salt) maize line and 2.63–27.40-fold in the salt-tolerant (T-salt) maize line. The remaining gene (*ZmWAK9*) exhibited opposite expression patterns under salt stress, it was downregulated by 43.51% in the salt-sensitive maize inbred line, but upregulated 1.03-fold in the salt-tolerant maize inbred line.

#### 3.9.3 Expression patterns of *ZmWAKs* under drought stress

The changes in the expression of *ZmWAKs* in response to drought stress were assessed using the transcriptome data (PRJNA576545) downloaded from the NCBI website (http://www.ncbi.nlm.nih.gov). A total of 34 *ZmWAKs* were found to be expressed in drought-tolerant and drought-intolerant hybrids under drought stress, among which six genes exhibited significantly high expression levels (TPM > 10) (**Supplementary Table S12**; **Figures 8E, F**). Among the highly expressed genes, three genes (*ZmWAK9*, *ZmWAK20*, and *ZmWAK45*) displayed similar expression patterns under drought stress, with downregulation by 13.40–21.5% in the drought-intolerant hybrid (ZX978) and by 23.40-41.05% in the drought-tolerant hybrid (ND476). The remaining three *ZmWAKs* (*ZmWAK2*, *ZmWAK35*, and *ZmWAK52*) displayed opposite expression patterns under drought stress, their expression levels were downregulated by 41.06–57.94% in the drought-intolerant hybrid and upregulated by 0.79–11.80% in the drought-tolerant hybrid.

#### 3.9.4 Expression patterns of *ZmWAKs* under waterlogging stress

The changes in the expression of *ZmWAKs* in response to waterlogging stress were assessed using previously published transcriptome data (PRJNA606824) (Yu et al., 2020). We observed that 43 *ZmWAKs* were expressed under waterlogging stress, among which 21 *ZmWAKs* displayed high expression levels (TPM > 10) (**Supplementary Table S13**; **Figures 8G, H**). Among the 21 genes, the expression levels of nine genes were consistently downregulated under waterlogging stress, while those of seven genes were consistently upregulated, compared to the waterlogging 0 hour (WS_0h). The expression levels of three genes (*ZmWAK22*, *ZmWAK38*, *ZmWAK43*) were upregulated at 2 or 4 hours under waterlogging stress (WS_2h, WS_4h), but subsequently downregulated compared to WS_0h. One gene (*ZmWAK42*) exhibited elevated expression levels at 4 and 10 hours under waterlogging stress (WS_4h, WS_10h), but lower expression levels at other time points. The remaining gene *ZmWAK41* was upregulated at all time points under waterlogging stress except at 8 hours, with a peak increase observed at 4 hours post-treatment. Under waterlogging stress, 11 *ZmWAKs* displayed the most significant changes. Among them, five genes (*ZmWAK9*, *ZmWAK15*, *ZmWAK25*, *ZmWAK34*, and *ZmWAK48*) were significantly downregulated by 85.97–99.04%, and four genes (*ZmWAK23*, *ZmWAK27*, *ZmWAK44*, and *ZmWAK45*) were significantly upregulated by 3.05–8.45-fold at WS_4h compared to WS_0h. The remaining two genes, *ZmWAK22* and *ZmWAK41*, showed peak expression levels at 4 hours under waterlogging stress, which were increased by 1.75-fold and 4.8-fold compared to WS_0h, respectively.

### 3.10 Expression patterns of *ZmWAKs* under biotic stresses

#### 3.10.1 Expression patterns of *ZmWAKs* under smut stress

**Figure 9:**
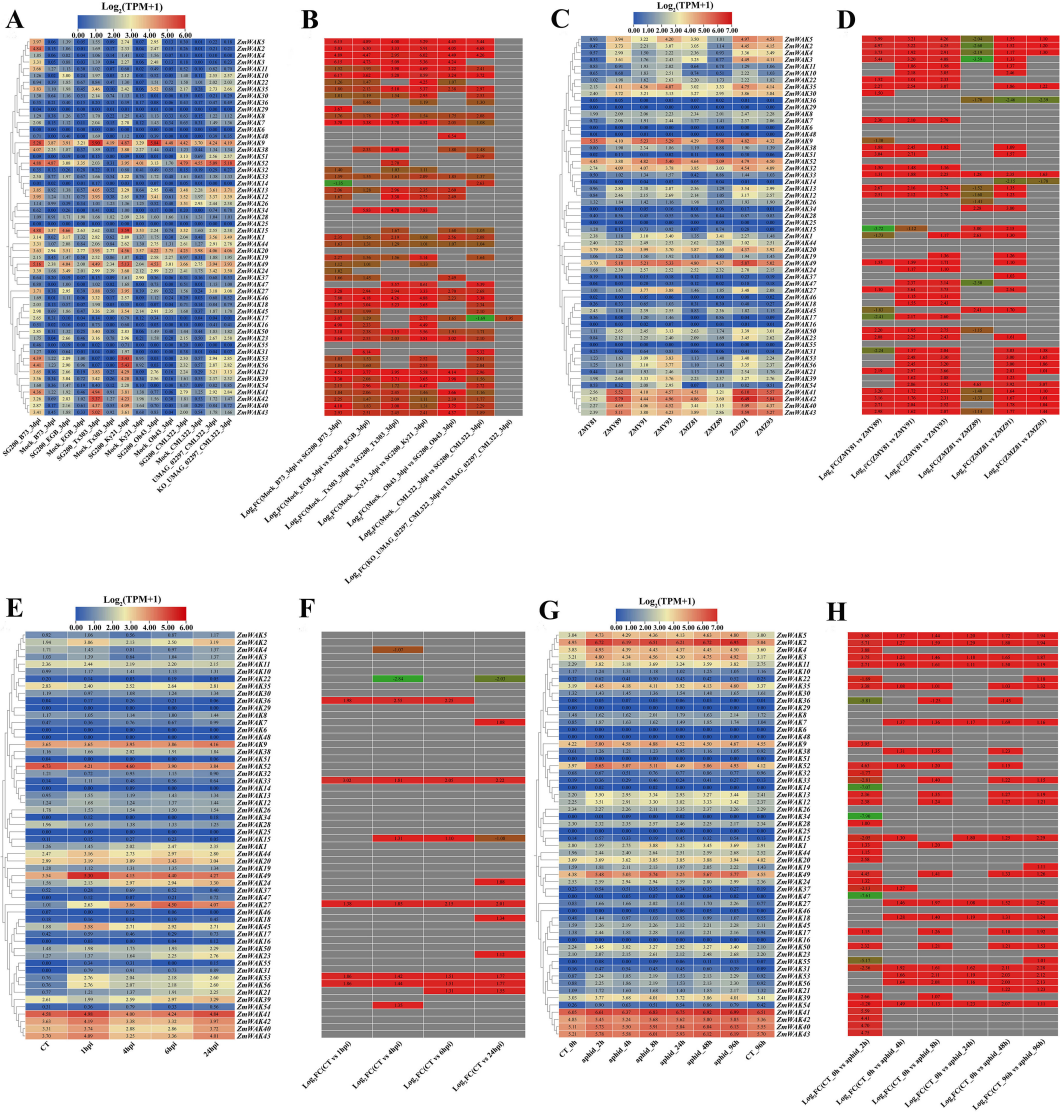
Maize *WAK* gene family expression heat map under biotic stress. **(A)** Expression pattern of maize *ZmWAK* genes under smut stress. SG 200 and UMAG_02297 are strains of the active vegetative fungus, which lead to maize smut. KO_UMAG_02297 is a mutant strain of UMAG_02297. Mock is the simulation control treatment involved uninfected plants. The term 3dpi refers to 3 days after infection. The six maize lines are B73, CML 322, EGB, Ky 21, Oh 43, and Tx 303. **(B)** Expression fold changes of the maize *WAK* gene family under smut stress. **(C)** Expression pattern of maize *ZmWAK* genes under gray leaf spot stress. ZMY represents the resistant inbreed line ‘Yayu 889’ and ZMZ represented the susceptible inbreed line ‘Zheng Hong 532’, they are sown in an field infected by *Mycosphaerella maydis* and the leaves of the infected plants are collected at 81, 89, 91, and 93 days after planting. **(D)** Expression fold changes of the maize *WAK* gene family under gray leaf spot stress. **(E)** Expression pattern of maize *ZmWAK* genes under beet armyworm stress. CT represents the uninfected plants, 1hpi represents 1 hour after infection, 4hpi represents 4 hours after infection, 6hpi represents 6 hours after infection, 24hpi represents 24 hours after infection. **(F)** Expression fold changes of the maize *WAK* gene family under beet armyworm stress. **(G)** Expression pattern of maize *ZmWAK* genes under aphid stress. CT_0h and CT_96h represent uninfected plants at 0 hour and 96 hours. aphid_2h, aphid_4h, aphid_8h, aphid_24h, aphid_48h, and aphid_96h represent infected plants at 2, 4, 8, 24, 48 and 96 hours, respectively. **(H)** Expression fold changes of the maize *WAK* gene family under aphid stress. There are two figures in each stress treatment—the left figure represents the log_2_(TPM + 1) and the right figure represents the log_2_ (Fold Change), indicated in red for up-regulation and green for down-regulation. When the absolute value of log_2_(Fold Change) is greater than 1, it will be shown in the figure.

The expression patterns of *ZmWAKs* under smut (*Sphacelotheca reiliana*) stress were analyzed using the transcriptome data (PRJNA673988) obtained from six maize inbred lines with distinct resistance levels (CML322 > B73 > EGB > Ky21 > Oh43 > T×303) (Schurack et al., 2021). Under smut stress, a total of 48 *ZmWAKs* were expressed in maize inbred lines (TPM > 1), with 25 genes displaying high expression levels (TPM > 10) (**Supplementary Table S14**; **Figures 9A, B**). Among the highly expressed genes, 23 *ZmWAKs* exhibited upregulation across all six maize inbreds following inoculation, except for *ZmWAK26* and *ZmWAK52*. The expression levels of 16 *ZmWAKs* (*ZmWAK2*, *ZmWAK5*, *ZmWAK11*, *ZmWAK12*, *ZmWAK13*, *ZmWAK21*, *ZmWAK27*, *ZmWAK35*, *ZmWAK39*, *ZmWAK40*, *ZmWAK41*, *ZmWAK42*, *ZmWAK43*, *ZmWAK49*, *ZmWAK53*, and *ZmWAK56*) exhibited the most significant changes under smut stress (TPM > 10, |Log_2_ (Fold Change)| > 1). Among them, 12, 10, 8, 2, 1, and 1 *ZmWAKs* were significantly upregulated under smut stress in the maize inbred lines B73, Ky21, T×303, oh43, EGB, and CML322, respectively. Further analysis revealed that *ZmWAK2*, *ZmWAK5*, and *ZmWAK11* were highly expressed exclusively in the resistant line B73, exhibiting respective upregulation of 255.19-, 396.26-, and 7.23-fold at 3 days post-infection.

#### 3.10.2 Expression patterns of *ZmWAKs* under gray leaf spot stress

The expression patterns of *ZmWAKs* under gray leaf spot (*Mycosphaerella maydis*) stress were analyzed using transcriptome data (PRJNA436207) obtained from a previous study (Yu et al., 2018). A total of 41 *ZmWAKs* were expressed in resistant (ZMY) and susceptible (ZMZ) maize cultivars (**Supplementary Table S15**; **Figures 9C, D**). Among them, 18 genes exhibited high expression levels (TPM > 10) and displayed significant expression variation under leaf spot stress (|Log_2_ (Fold Change)| > 1). Further analysis showed that five genes (*ZmWAK27*, *ZmWAK35*, *ZmWAK39*, *ZmWAK53*, and *ZmWAK56*) were upregulated in both resistant and susceptible cultivars under gray leaf spot stress, compared to plants at 81 days post-infection. Twelve genes (*ZmWAK2*, *ZmWAK3*, *ZmWAK4*, *ZmWAK5*, *ZmWAK13*, *ZmWAK30*, *ZmWAK32*, *ZmWAK40*, *ZmWAK41*, *ZmWAK42*, *ZmWAK43*, and *ZmWAK49*) were upregulated in the resistant cultivar, while in the susceptible cultivar, these genes were initially downregulated at 89 days post-infection and subsequently upregulated. One gene (*ZmWAK9*) showed opposite expression patterns in resistant and susceptible cultivars, its expression level was downregulated by 4.59–59.42% in the resistant cultivar and upregulated by 1.74–76.31% in the susceptible cultivar.

#### 3.10.3 Expression patterns of *ZmWAKs* under beet armyworm stress

The expression patterns of *ZmWAKs* under beet armyworm (*Spodoptera exigua*) stress were analyzed using maize leaf transcriptome data (PRJNA625224) downloaded from the NCBI website (http://www.ncbi.nlm.nih.gov). We detected a total of 36 *ZmWAKs*, with nine genes exhibiting relatively high expression levels (TPM > 10) (**Supplementary Table S16**; **Figures 9E, F**). Further analysis revealed that three *ZmWAKs* (*ZmWAK27*, *ZmWAK45*, and *ZmWAK49*) were consistently upregulated, and one gene (*ZmWAK52*) was consistently downregulated under beet armyworm stress. Four *ZmWAKs* (*ZmWAK40*, *ZmWAK41*, *ZmWAK42*, and *ZmWAK43*) exhibited biphasic expression patterns, being upregulated at 1 hour and 24 hours after infestation but downregulated at 4 hours and 6 hours after infestation. One gene (*ZmWAK9*) displayed a slight downregulation at 1 hour after infestation, followed by an increase in expression. Among these genes, *ZmWAK27* displayed the most significant expression variation, exhibiting 4.09–20.32-fold upregulation after infestation.

#### 3.10.4 Expression patterns of *ZmWAKs* under aphid stress

The expression pattern of *ZmWAKs* under aphid (*Rhopalosiphum maidis*) stress was analyzed using the transcriptome data (PRJNA295410) of maize foliar responses to aphid feeding obtained from a previous study (Tzin et al., 2015). A total of 38 *ZmWAKs* were expressed in maize foliar tissue, with 18 genes displaying high expression levels (**Supplementary Table S17**; **Figures 9G, H**). Among them, the expression levels of 16 genes were consistently upregulated under aphid stress. One gene (*ZmWAK1*) was downregulated at 2 and 4 hours after infestation, but was subsequently upregulated. The remaining gene, *ZmWAK20*, exhibited a dynamic expression pattern characterized by downregulation at 2, 4, and 96 hours after infestation and upregulation at 8, 24, and 48 hours after infestation. Further analysis revealed that 12 *ZmWAKs* showed the most significant expression variations (|Log_2_ (Fold Change)| >1). Among them, the expression of five genes (*ZmWAK4*, *ZmWAK11*, *ZmWAK12*, *ZmWAK13*, and *ZmWAK52*) peaked at 2 hours after infestation, with expression levels increasing by 1.22- to 2.37-fold. The expression of one gene (*ZmWAK1*) peaked at 8 hours after infestation, with expression level rising by 1.29-fold. The remaining six genes (*ZmWAK2*, *ZmWAK3*, *ZmWAK5*, *ZmWAK35*, *ZmWAK39*, and *ZmWAK49*) peaked at 96 hours after infestation, with expression levels increasing by 0.57- to 2.83-fold.

### 3.11 Expression patterns of *ZmWAKs* under hormonal treatments

**Figure 10:**
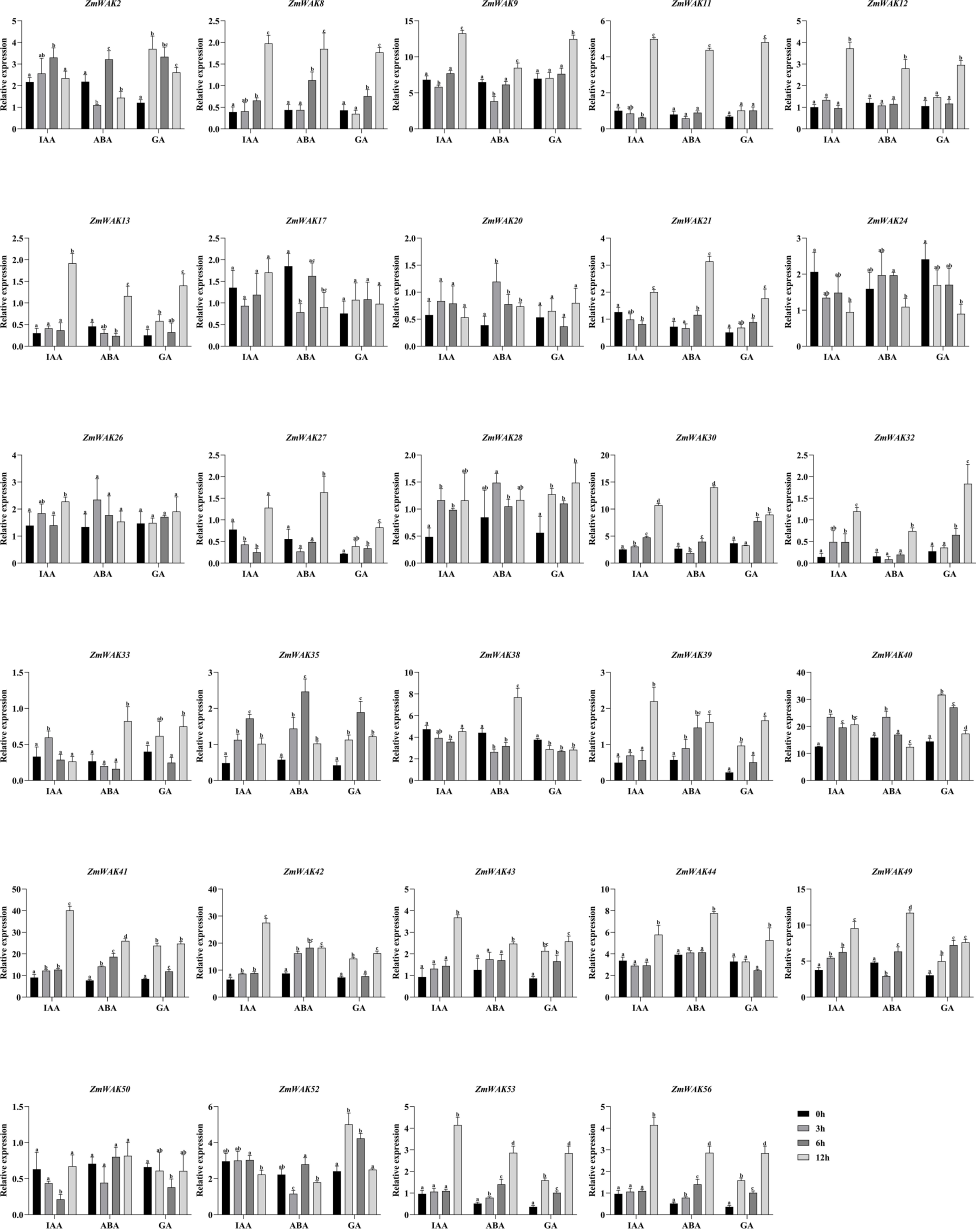
The expression pattern of *WAK* genes in maize under hormonal treatments. The black column represents 0 hour after hormonal treatments. The light gray column represents 3 hours after hormonal treatments. The dark gray column represents 6 hours after hormonal treatments. The gray column represents 12 hours after hormonal treatments. The level of significance (p < 0.05) among the different treatments is indicated by different letters.

RNA-seq was performed to explore the expression patterns of *ZmWAKs* under IAA, ABA, and GA treatments. A total of 29 *ZmWAKs* were detected to be expressed in treated maize leaf tissue (FPKM > 1; **Supplementary Table S18**; **Figure 10**). Under IAA treatment, the expression levels of 26 *ZmWAKs* significantly varied. Among them, the levels of three, two, and 14 genes peaked at 3, 6, and 12 hours post-treatment, respectively. Three genes showed the lowest expression levels at 3 or 6 hours post-treatment. The remaining four genes displayed a significant downregulation at 3 or 6 hours post-treatment, followed by a significant upregulation. Under ABA treatment, the expression levels of 26 *ZmWAKs* varied significantly. Among them, the levels of one, two, and 13 genes peaked at 3, 6, and 12 hours post-treatment. Three genes were significantly downregulated at 3 hours post-treatment. The levels of six genes significantly decreased, followed by a subsequent increase. In contrast, one gene showed an opposite expression pattern by initially upregulating and then downregulating. Under GA treatment, the levels of 26 genes varied significantly. Among them, the levels of three, one, and 19 genes peaked at 3, 6, and 12 hours post-treatment. Three genes significantly downregulated at 6 or 12 hours post-treatment. Overall, six genes (*ZmWAK9*, *ZmWAK30*, *ZmWAK40*, *ZmWAK41*, *ZmWAK42*, and *ZmWAK49*) displayed the highest expression and the most significant variations under IAA, ABA, and GA treatments, upregulating by 0.88–3.42-fold, 0.79–1.95-fold, and 0.30–4.26 fold, respectively.

### 3.12 Analysis of differentially expressed *ZmWAKs* under abiotic, biotic, and hormonal treatments

The expression profiles of maize *WAK* genes were analyzed under various abiotic, biotic, and hormonal treatments and heat maps were generated to visualize the differentially expressed genes (**Supplementary Table S19**, **Supplementary Figure S2**). Overall, the expression of 34 *ZmWAKs* significantly varied in response to abiotic, biotic, and hormonal treatments. Further analysis revealed that *ZmWAK10*, *ZmWAK15*, *ZmWAK20*, *ZmWAK22*, *ZmWAK23*, *ZmWAK24*, *ZmWAK25*, *ZmWAK34*, *ZmWAK44*, and *ZmWAK48* were differentially expressed under abiotic stresses. Conversely, *ZmWAK5*, *ZmWAK11*, *ZmWAK12*, *ZmWAK13*, *ZmWAK32*, *ZmWAK43*, *ZmWAK53*, and *ZmWAK56* were differentially expressed under biotic stresses. Additionally, *ZmWAK1*, *ZmWAK2*, *ZmWAK3*, *ZmWAK4*, *ZmWAK9*, *ZmWAK21*, *ZmWAK27*, *ZmWAK30*, *ZmWAK35*, *ZmWAK39*, *ZmWAK40*, *ZmWAK41*, *ZmWAK42*, *ZmWAK45*, *ZmWAK49*, and *ZmWAK52* were differentially expressed under multiple stresses. Among these genes, the expression of *ZmWAK9*, *ZmWAK27*, *ZmWAK41*, and *ZmWAK49* varied significantly under more than six types of stresses.

### 3.13 Analysis of the maize WAK protein interaction network

To further explore the function of the maize *WAK* gene family, an interaction network of maize WAK proteins was predicted using the STRING database. Our results showed that the maize *WAK* genes interacted with several other genes, including *Cl3398_1*, *IDP4123*, *LOC100384169*, *K7U0Q4*, *LAZ1-8*, *ATL46-5*, and *YLS9* (**Supplementary Figure S3**). *Cl3398_1* encodes a cAGP10-like protein, which displays homology to canonical arabinogalactan protein 10 (AGP10). AGP10 anchors in the plasma membrane and plays crucial roles in cell recognition, cell wall extension, and signal transduction. *IDP4123* encodes a pollen Ole 1 allergen and extensin family protein (POE), which functions as developmental regulators in several plant tissues (Hu et al., 2014). *LOC100384169* encodes a putative 3-deoxy-D-manno-octulosonic acid transferase (KDTA), which localizes in the mitochondria and is involved in the synthesis of a mitochondrial lipid A-like molecule (Lerouge, 2010). *K7U0Q4* encodes germin-like protein subfamily 1 member 8, which is differentially expressed under salt stress (Soares et al., 2018). *LAZ1–8* encodes a protein with a DUF300 domain, which positively regulates systemic acquired resistance (SAR) via the regulation of the expression of *CBP60g*, *SARD1*, and SA and BHP biosynthetic genes (Chen et al., 2024). *ATL46–5* and *YLS9* encode RING-type domain-containing protein and late embryogenesis abundant protein, respectively.

## 4 Discussion

In this study, a total of 56 *ZmWAKs* were identified in the maize genome (**Supplementary Table S1**). Phylogenetic analysis showed that the maize *WAK* gene family members can be classified into seven subgroups (**Figure 3**). In *Arabidopsis*, rice, soybean, and sorghum, the *WAK* gene families comprise 27, 125, 74, and 98 *WAKs*/*WAKLs*, respectively (He et al., 1999; Verica and He, 2002; Zhang et al., 2005; Li et al., 2025; Whyte and Sipahi, 2024). This finding revealed that the number of the *WAK* gene family members significantly varies among different plant species. Maize *WAK* genes in the same subgroups share similar gene structures and conserved motifs, implying that they might mediate similar functions in plants (**Supplementary Table S1**; **Figure 4**).

*ZmWAK1* and *ZmWAK15*, classified in subgroup II, are closely related to *AtWAKL14* and *AtWAKL21*. In *Arabidopsis*, *AtWAKL14* interacts with pectin and oligogalacturonides (Ogs) and regulates vascular tissue development (Ma et al., 2024). *ZmWAK27*, classified in subgroup III, is closely related to *AtWAKL20*. *AtWAKL20* is significantly downregulated under ABA and JA treatments (by 63.1% and 84.5%, respectively) (Chae et al., 2009). A T-DNA mutant of *AtWAKL20* revealed that *atwakl20* mutation did not affect root gravitropism (Han et al., 2024). In addition, a total of 13 *ZmWAKs* (*ZmWAK2*, *ZmWAK3*, *ZmWAK4*, *ZmWAK5*, *ZmWAK7*, *ZmWAK8*, *ZmWAK10*, *ZmWAK11*, *ZmWAK22*, *ZmWAK29*, *ZmWAK35*, *ZmWAK36*, and *ZmWAK48*) from subgroup V and four *AtWAKLs* (*AtWAKL11*, *AtWAKL13*, *AtWAKL17*, and *AtWAKL18*) are clustered together. *AtWAKL11* interacts with *NAC102*, which is involved in modulating cell wall pectin metabolism and the binding of cadmium (Cd) to the cell wall, thereby enhancing Cd tolerance in *Arabidopsis* (Han et al., 2023). Two *ZmWAKs* (*ZmWAK6* and *ZmWAK30*) from subgroup VII group together with seven *AtWAKs*/*WAKLs* (*AtWAK1*, *AtWAK2*, *AtWAK3*, *AtWAK4*, *AtWAK5*, *AtWAKL12*, and *AtWAKL16*). The overexpression of *AtWAK1* had been shown to enhance resistance to *Botrytis cinerea* in transgenic plants (Brutus et al., 2010). In a previous study, expression of the dominant hyperactive allele for *AtWAK2* was found to lead to a dwarf phenotype and activation of stress response and reactive oxygen species (ROS) production (Kohorn et al., 2021). The antisense expression of *AtWAK4* had been found to inhibit cell elongation and alter cellular morphology (Lally et al., 2001). In the current study, the promoter regions of *ZmWAK6* and *ZmWAK30* are found to harbor several elements related to MeJA and JA stresses, defense responses, and meristem expression (**Supplementary Table S4**). MeJA and JA are important regulators of plant responses to biotic and abiotic stresses as well as plant development (Wasternack and Hause, 2013). Thus, *ZmWAK6* and *ZmWAK30* might be involved in plant growth, development, and stress responses via the MeJA and JA signaling pathways.

Gene duplication events, including SDs and TDs, have been recognized as predominant forces driving the expansion of gene families (Cannon et al., 2004). The analysis of gene duplication within the maize *WAK* gene family reveals that a total of nine SD and two TD events have contributed to the expansion of this gene family, collectively accounting for 23.2% of *ZmWAKs* (**Table 1**). In soybean, 37.83%, 20.27%, and 12.16% of *GmWAKs* are produced by SD, TD, and proximal duplication events, respectively (Zhang et al., 2005). In cotton, 62.07%, 17.24%, and 17.24% of *GhWAKs* are produced by SD, TD, and proximal duplication events, respectively (Dou et al., 2021). These results indicate that whole genome duplication (WGD) events, particularly SD events, play significant roles in the expansion of the *WAK* gene families of several plant species.

During whole genome duplication (WGD), the accumulated base mutations might facilitate the emergence of genes undergoing neofunctionalization or subfunctionalization (Roulin et al., 2013). Neofunctionalization gives rise to novel functional genes, which usually undergoes positive selection (Ka/Ks > 1) (Conant et al., 2014). Subfunctionalization leads to the function partitioning of the ancestral genes that experience purifying selection (Ka/Ks < 1) (Lynch and Conery, 2000). In the current study, the Ka/Ks analysis of 11 duplication gene pairs show that four gene pairs exhibit a Ka/Ks ratio of >1, suggesting that these genes have undergone positive selection and might have diversified to acquire new functions (**Table 1**). The remaining seven gene pairs have a Ka/Ks ratio of <1, indicating that they might have experienced purifying selection, leading to the subfunctionalization of the genes.

The interspecies collinearity analysis (**Figure 6**) of the *WAK* gene families from *Arabidopsis*, maize, soybean, rice, and sorghum demonstrates that 35 maize *WAK* genes exhibits collinearity with the *WAK* genes of soybean, rice, and sorghum, demonstrating a total of 76 collinear relationships. Maize *WAK* genes exhibits eight, 27, and 41 collinear relationships with soybean, rice, and sorghum *WAKs*, respectively, with *ZmWAK37* showing the most collinear relationships (n=6), and *ZmWAK11*, *ZmWAK14*, *ZmWAK44*, *ZmWAK47*, and *ZmWAK49* showing four collinear relationships each. However, none of the *ZmWAKs* exhibit collinear relationships with *Arabidopsis WAKs* (**Supplementary Table S6**, **Figure 6**). The SD and TD events of maize *WAKs* were predicted to have occurred around 2.55–226.02 million years ago, with maize diverging from rice and sorghum approximately 227.56 and 211.68 million years ago, respectively (**Table 1**). These results imply that maize exhibited higher homology with the monocotyledonous plants (rice and sorghum) than with the dicotyledonous plants (*Arabidopsis* and soybean).

Tissue-specific expression of maize *WAK* gene family members was analyzed using publicly available transcriptome data. Maize *WAK* gene family exhibits distinct expression patterns (**Supplementary Tables S7**–**S9**). *ZmWAK13*, *ZmWAK15*, *ZmWAK22*, *ZmWAK25*, *ZmWAK43*, and *ZmWAK49* were highly expressed in the root. *ZmWAK2*, *ZmWAK9*, *ZmWAK15*, *ZmWAK35*, *ZmWAK49*, and *ZmWAK52* were highly expressed in the leaf. *ZmWAK9* and *ZmWAK15* were highly expressed in the seed during the early growth stage. Among these genes, *ZmWAK9*, *ZmWAK15*, and *ZmWAK49* were highly expressed in multiple tissues, including root, leaf, and seed. The expression of *ZmWAK9* peaked in the V9 eighth leaf and the seed in its early growth stage. *ZmWAK49* levels peaked in the Z4 region of the taproot at 7 days post-sowing and in the V9 thirteenth leaf. *ZmWAK15* was highly expressed in the root, stem, and SAM at the V3 stage, in the first elongated internode at the V5 stage, in the V9 eleventh and thirteenth leaves, and in seeds during the early development stages.

*Cis*-acting element analysis demonstrated that the promoter regions of *ZmWAK9*, *ZmWAK15*, and *ZmWAK49* contained MeJA- and JA-responsive elements (TGACG-motif, as-1, and CGTCA-motif), IAA-responsive elements (TGA-element and TGA-box), as well as meristem expression-related elements (CAT-box and CCGTCC motif; **Supplementary Table S4**). This result implies that these genes might participate in the regulation of plant growth and development by responding to MeJA, JA, and IAA.

The analysis of stress response patterns of maize *WAK* gene family showed that the expression of *ZmWAK9*, *ZmWAK27*, *ZmWAK41*, and *ZmWAK49* significantly varied under different abiotic, biotic, or hormonal treatments (**Figures 8**-**10**). *ZmWAK9* was downregulated under drought, waterlogging, and gray leaf spot stresses but upregulated under beet armyworm, IAA, ABA, and GA treatments. Under salt stress, *ZmWAK9* displayed opposite expression patterns in the salt-tolerant (T-salt) and salt-sensitive (S-salt) maize inbred lines, with upregulation under T-salt and downregulation under S-salt. *ZmWAK27* was significantly downregulated under temperature stress but upregulated under salt, waterlogging, smut, leaf spot, and beet armyworm stresses. *ZmWAK41* was significantly induced by waterlogging, smut, gray leaf spot, beet armyworm, IAA, ABA, and GA treatments. In addition, *ZmWAK49* was robustly induced by salt, smut, gray leaf spot, beet armyworm, aphid, IAA, ABA, and GA treatments.

In summary, the expression of *ZmWAK9*, *ZmWAK15*, *ZmWAK27*, *ZmWAK41*, and *ZmWAK49* significantly varied during plant growth and development and under different stress conditions (**Supplementary Tables S7**-**S18**). *ZmWAK9* encodes receptor-like protein kinase rlk13, which localizes on the plasma membrane and is involved in the cell surface receptor signaling pathway and protein phosphorylation. *ZmWAK9* was found to be homologous to the rice *OsWAK53* (*Os04g0599000*), with a homology of 52.91% (**Figure 6**). *OsWAK53* had been identified as an upstream gene of *OsPID*, and they participated in the adaptation to low-phosphorus stress by maintaining chlorophyll content in rice leaves (Zhou, 2022). In the present study, *ZmWAK9* was highly expressed in the V9 eighth leaf and during the early stages of seed development. It was significantly upregulated under armyworm, IAA, ABA, and GA treatments. Under salt stress, *ZmWAK9* was upregulated by 103.1% in salt-tolerant material but downregulated by 43.5% in salt-sensitive material. This finding suggests that *ZmWAK9* might be involved in the regulation of plant growth and development and responses to multiple stresses via hormone signaling pathways.

*ZmWAK15* was significantly upregulated by 1.61–5.67-fold under smut stress compared to the control. A previous study reported that the pathogen *Sporisorium reilianum* activated *ZmWAK15* (Zhang et al., 2024). This activation triggered the *WAK-SnRK1a2-WRKY53* module, which led to the downregulation of genes associated with transmembrane transport and carbohydrate metabolism. Consequently, the fungus experienced nutrient starvation in the intercellular spaces, resulting in enhanced resistance to smut disease in maize (Zhang et al., 2024). In the present study, *ZmWAK15* was highly expressed in multiple tissues of maize, including the root, stem, SAM, leaf, and seed, with the highest expression level observed in the roots. This finding shows that *ZmWAK15* is involved not only in maize smut resistance but also in the regulation of maize growth and development. It suggests that *ZmWAK15* is one of the most important members in maize *WAK* gene family and plays an important role in maize.

*ZmWAK27* encodes a WAK-related receptor-like protein kinase. *ZmWAK27* and *AtWAKL20* fall within the same evolutionary clade, sharing a homology of 49.12%. Under ABA and JA treatments, The expression of *AtWAKL20* was significantly suppressed (Chae et al., 2009). *AtWAKL20* had been found to negatively regulate the immune response by phosphorylating the Ser470 residue in the NB-ARC domain of BSR1 (Zhong et al., 2025). In the current study, under temperature stress, *ZmWAK27* was markedly downregulated by 78.3–95.78%. In contrast, under salt stress, *ZmWAK27* was considerably upregulated by 5.31- and 9.39-fold in salt-sensitive and -tolerant inbred lines, respectively. Furthermore, under waterlogging, smut, gray leaf spot, and beet armyworm stresses, *ZmWAK27* was upregulated by 50.67–3.52-fold, 12.08–45.48-fold, 0.72–12.55-fold, and 4.07–20.26-fold, respectively. Thus, *ZmWAK27* can be induced by various stresses, suggesting its involvement in the responses to both biotic and abiotic stresses.

*ZmWAK41* encodes a serine/threonine receptor-like kinase, which is homologous to rice *RLG2* with a sequence similarity of 67.27%. Rice *RLG2* encodes the rust resistance kinase Lr10, which was initially discovered in wheat and plays a crucial role in the plant’s defense mechanism against leaf rust pathogens (Loutre et al., 2009; Kurt and Yagdi, 2021). *ZmWAK41* was substantially upregulated by 4.79-fold at 4 hours after waterlogging treatment. Under smut and gray leaf spot stresses, *ZmWAK41* was considerably upregulated by 6.34–33.1-fold and 0.67–12.79-fold compared to the control, respectively. At 1 and 24 hours after beet armyworm treatment, *ZmWAK41* was upregulated by 33.85% and 20.77%, respectively. Furthermore, under IAA, ABA, and GA treatments, *ZmWAK41* was significantly upregulated by 1.94–3.42-fold compared to the control. The marked inductions of *ZmWAK41* under waterlogging, smut, gray leaf spot, beet armyworm, IAA, ABA, and GA treatments indicate its potential role in mediating resistance to both biotic and abiotic stresses.

*ZmWAK49* is homologous to *Arabidopsis AtLRK10L1* (AT1G18390), with an amino acid sequence homology of 37.98%. *AtLRK10L1* generates two different transcripts, *AtLRK10L1.1* and *AtLRK10L1.2*, under the action of two different promoters (Shin et al., 2015). *At-LRK10L1.2* is involved in ABA-mediated signaling. Its T-DNA insertion mutant exhibites reduced sensitivity to ABA but increased sensitivity to drought stress (Lim et al., 2015). We find that *ZmWAK49* exhibites synteny with the rice gene *LOC_Os05g47770* (**Supplementary Table S6**). In a previous study, *LOC_Os05g47770* had been identified as the candidate gene associated with salt tolerance during the flowering stage of rice (Lekklar et al., 2019). At 24 and 72 hours post-inoculation of *Magnaporthe oryzae*, the expression of *LOC_Os05g47770* in the near-isogenic lines, which carried resistance genes, were significantly upregulated by 4.5- and 10.49-fold, respectively (Jain et al., 2019). *ZmWAK49* was highly expressed in the Z4 region of root tips at 7 days after sowing and in the V9 thirteenth leaf. Under salt stress, *ZmWAK49* was upregulated by 0.59-fold in the salt-sensitive inbred line and by 2.63-fold in the salt-tolerant inbred line. Moreover, under smut, gray leaf spot, beet armyworm, and aphid stresses, *ZmWAK49* was upregulated by 1.03–10.0-fold, 0.63–3.79-fold, 0.72–2.6-fold, and 0.6–1.7-fold, respectively. Furthermore, *ZmWAK49* levels peaked at 24 hours post-treatment with IAA, ABA, and GA treatments, upregulating by 2.54-, 2.44-, and 2.51-fold compared to the control, respectively. Thus, *ZmWAK49* was significantly induced by various hormones and stresses, suggesting that *ZmWAK49* might contribute to plant development and stress responses via hormone signaling pathways.

## 5 Conclusion

In this study, a total of 56 *ZmWAKs* were identified in the maize genome. Based on chromosomal location, phylogenetic tree, gene structure, and conserved motif analyses, the identified genes were divided into seven subgroups, with 54 genes being mapped to maize chromosomes. Every subgroup displayed an analogous exon-intron structure and conserved motifs. Gene duplication and collinearity analyses revealed that the maize *WAK* gene family experienced SD and TD events. Among the duplication gene pairs, four pairs experienced positive selection, whereas seven pairs experienced purifying selection. A high degree of collinearity was observed between maize and monocotyledonous plants, such as rice and sorghum. Transcriptome data analysis showed that the expression patterns of *ZmWAK* in maize were distinct in various plant tissues and under different biotic and abiotic stresses. Notably, *ZmWAK9*, *ZmWAK15*, *ZmWAK27*, *ZmWAK41*, and *ZmWAK49* were simultaneously and significantly induced by multiple stresses, implying that they might be crucial for maize growth and development as well as stress responses. Our results provided valuable insights into the function and evolution of maize *WAKs*, revealing potential candidate genes for stress tolerance research in maize.

## Data Availability

The original contributions presented in the study are included in the article/**Supplementary Material**. The transcriptome data from the hormonal treatment of maize reported in this study have been deposited in the National Center for Biotechnology Information (NCBI) under the BioProject accession number PRJNA1309972.

## Supplementary material

Supplementary Figure 1 GO annotation analysis of *ZmWAK*s in maize genome. MF indicates molecular function. CC indicates cellular component. BP indicates biological process. Red dot plots indicate the most genes involved in that process, while small green plots indicate the least genes involved.

Supplementary Figure 2 The expression pattern of *WAK* genes in maize under abiotic, biotic and hormonal treatments. Expression analysis was conducted on genes with high expression levels (TPM > 10) and the most significant variation under different stresses. For each gene, the expression value exhibiting the maximum variation (log_2_(Fold Change)) under each stress category was selected as the representative data for heatmap construction. Temperature represents the temperature stress. S_Salt and T_Salt represent the salt-stress sensitive and tolerant materials under salt stress, respectively. Drought_intolerant and Drought _tolerant represent the drought-stress intolerant and tolerant materials under drought stress, respectively. Waterlogging represents the waterlogging stress. Smut disease represents the smut stress. Leaf spot represents the gray leaf spot stress. Armyworm represents the beet armyworm stress. Aphid represents the aphid stress. IAA, ABA and GA represent the IAA, ABA, and GA treatments, respectively. The red color represents an escalation in expression, the green color represents a reduction in expression.

Supplementary Figure 3 The function interaction network of maize WAK proteins. **(A)** The maize WAK protein interaction network constructed via homology modeling based on *Arabidopsis thaliana* proteins. **(B)** The maize WAK protein interaction network constructed via homology modeling based on rice proteins.

Supplementary Table 1 Physicochemical parameters of 56 *ZmWAK* genes in maize genome.

Supplementary Table 2 Conserved motif information in ZmWAK proteins. A total of 10 motifs are shown, and their E-values and sequence with are provided. The height of each letter represents the probability of amino acids at the respective position.

Supplementary Table 3 Protein motif structure analysis of *ZmWAK* genes.

Supplementary Table 4
*Cis*-acting elements in promoter regions of ZmWAK genes in maize.

Supplementary Table 5 Function information of the *cis*-acting elements in the promoters of *ZmWAK* genes.

Supplementary Table 6 Collinearity analysis of the *WAK* family genes among maize, *Arabidopsis*, soybean, rice and sorghum.

Supplementary Table 7 Expression profiles of *ZmWAK* genes during root development.

Supplementary Table 8 Expression profiles of *ZmWAK* genes during leaf development.

Supplementary Table 9 Expression profiles of *ZmWAK* genes during seed development.

Supplementary Table 10 Expression pattern of maize *ZmWAK* genes under temperature stress.

Supplementary Table 11 Expression pattern of maize *ZmWAK* genes under salt stress.

Supplementary Table 12 Expression pattern of maize *ZmWAK* genes under drought stress.

Supplementary Table 13 Expression pattern of maize *ZmWAK* genes under waterlogging stress.

Supplementary Table 14 Expression pattern of maize *ZmWAK* genes under smut stress.

Supplementary Table 15 Expression pattern of maize *ZmWAK* genes under gray leaf spot stress.

Supplementary Table 16 Expression pattern of maize *ZmWAK* genes under beet armyworm stress.

Supplementary Table 17 Expression pattern of maize *ZmWAK* genes under aphid stress.

Supplementary Table 18 Expression pattern of maize *ZmWAK* genes under the hormonal treatment.

Supplementary Table 19 Expression patterns of maize *ZmWAK* genes under abiotic, biotic and hormonal treatments.

## References

[B1] Abedi A. Hajiahmadi Z. Kordrostami M. Esmaeel Q. Jacquard C. (2021). Analyses of lysin-motif receptor-like kinase (*LysM-RLK*) gene family in allotetraploid *Brassica napus* L. and its progenitor species: an in silico study. Cells. 11 (1), 37. doi: 10.3390/cells11010037, PMID: 35011598 PMC8750388

[B2] Al-Bader N. Meier A. Geniza M. Gongora Y. S. Jaiswal P. (2019). Loss of premature stop codon in the *wall-associated kinase* 91 (*oswak91*) gene confers sheath blight disease resistance in rice. BioRxiv. 625509. doi: 10.1101/625509 PMC10530950 37761813

[B3] Bai W. Li J. Zhang D. Sun F. Niu Y. Wang P.. (2024). A tyrosine kinase-like gene *BdCTR1* negatively regulates flowering time in the model grass plant *Brachypodium distachyon* . J. Plant Growth Regul. 43, 4577–4587. doi: 10.1007/s00344-024-11418-4

[B4] Bot P. Mun B. G. Imran Q. M. Hussain A. Lee S. U. Loake G.. (2019). Differential expression of *ATWAKL10* in response to nitric oxide suggests a putative role in biotic and abiotic stress responses. Peer J. 7, e7383. doi: 10.7717/peerj.7383, PMID: 31440429 PMC6699482

[B5] Brutus A. Sicilia F. Macone A. Cervone F. De Lorenzo. G. (2010). A domain swap approach reveals a role of the plant wall-associated kinase 1 (*WAK1*) as a receptor of oligogalacturonides. Proc. Natl. Acad. Sci. U.S.A. 107, 9452–9457. doi: 10.1073/pnas.1000675107, PMID: 20439716 PMC2889104

[B6] Cannon S. B. Mitra A. Baumgarten A. Young N. D. May G. (2004). The roles of segmental and tandem gene duplication in the evolution of large gene families in *Arabidopsis thaliana* . BMC Plant Biol. 4, 10. doi: 10.1186/1471-2229-4-10, PMID: 15171794 PMC446195

[B7] Chae L. Sudat S. Dudoit S. Zhu T. Luan S. (2009). Diverse transcriptional programs associated with environmental stress and hormones in the Arabidopsis receptor-like kinase gene family. Mol. Plant. 2, 84–107. doi: 10.1093/mp/ssn083, PMID: 19529822 PMC2639733

[B8] Chen Y. Han Y. Huang W. Zhang Y. Chen X. Li D.. (2024). Lazarus 1 functions as a positive regulator of plant immunity and systemic acquired resistance. Front. Plant Sci. 20. doi: 10.3389/fpls.2024.1490466, PMID: 39634069 PMC11614604

[B9] Chen C. Wu Y. Li J. Wang X. Zeng Z. Xu J.. (2023). TBtools-II: A “one for all, all for one” bioinformatics platform for biological big-data mining. Mol. Plant. 16, 1733–1742. doi: 10.1016/j.molp.2023.09.010, PMID: 37740491

[B10] Chen S. Zhou Y. Chen Y. Gu J. (2018). Fastp: an ultra-fast all-in-one FASTQ preprocessor. Bioinformatics. 34, i884–i890. doi: 10.1093/bioinformatics/bty560, PMID: 30423086 PMC6129281

[B11] Conant G. C. Bircher J. A. Pires J. C. (2014). Dosage, duplication and diploidization: clarifying the interplay of multiple models for duplicate gene evolution over time. Curr. Plant Biol. 19, 91–98. doi: 10.1016/J.PBI.2014.05008, PMID: 24907529

[B12] Dai Z. Pi Q. Liu Y. Hu L. Li B. Zhang B.. (2024). *ZmWAK02* encoding an RD-WAK protein confers maize resistance against gray leaf spot. New Phytologist. 241, 1780–1793. doi: 10.1111/nph.19465, PMID: 38058244

[B13] Decreux A. Messiaen J. (2005). Wall-associated kinase *WAK1* interacts with cell wall pectins in a calcium-induced conformation. Plant Cell Physiol. 46, 268–278. doi: 10.1093/pcp/pci026, PMID: 15769808

[B14] Dou L. L. Li Z. F. Shen Q. Shi H. R. Li H. Z. Wang W. B.. (2021). Genome-wide characterization of the *WAK* gene family and expression analysis under plant hormone treatment in cotton. BMC Genomics. 22, 85. doi: 10.1186/s12864-021-07378-8, PMID: 33509085 PMC7842020

[B15] Gao S. Zhang Z. Zhao Y. Li X. Wu Y. Huo W.. (2024). Cell wall-associated receptor kinase *GbWAKL26* positively regulate salt tolerance by maintaining Na^+^ and K^+^ homeostasis in cotton. Environ. Exp. Bot. 226, 105926. doi: 10.1016/j.envexpbot.2024.105926

[B16] Han E. Geng Z. Qin Y. Wang Y. Ma S. (2024). Single-cell network analysis reveals gene expression programs for *Arabidopsis* root development and metabolism. Plant Commun. 5, 100978. doi: 10.1016/j.sxplc.2024.100978, PMID: 38783601 PMC11369779

[B17] Han G. H. Huang R. N. Hong L. H. Xu J. X. Hong Y. G. Wu Y. H.. (2023). The transcription factor *NAC102* confers cadmium tolerance by regulating *WAKL11* expression and cell wall pectin metabolism in *Arabidopsis* . J. Integr. Plant Biol. 65, 2262–2278. doi: 10.1111/jipb.13557, PMID: 37565550

[B18] He Z. H. Cheeseman I. He D. Kohorn B. D. (1999). A cluster of five cell wall-associated receptor kinase genes, *WAK1-5*, are expressed in specific organs of *Arabidopsis* . Plant Mol. Biol. 39, 1189–1196. doi: 10.1023/A:1006197318246, PMID: 10380805

[B19] He Z. Zhang H. Gao S. Lercher M. J. Chen W. H. Hu S. (2016). Evolview v2: an online visualization and management tool for customized and annotated phylogenetic trees. Nucleic Acids Res. 44, W236–W241. doi: 10.1093/nar/gkw370, PMID: 27131786 PMC4987921

[B20] Hu Z. (2023). The functional study of wall-associated receptor kinase *OsWAK12* in regulating rice root development. (Zhengzhou, China: Henan Agriculture University). doi: 10.27117/d.cnki.ghenu.2023.000383

[B21] Hu K. Dai Q. Ajayo B. S. Wang H. Hu Y. Li Y.. (2023). Insights into *ZmWAKL* in maize kernel development: genome-wide investigation and ga-mediated transcription. BMC Genomics. 24, 15. doi: 10.1186/s12864-023-09849-6, PMID: 38082218 PMC10712088

[B22] Hu B. Liu B. Liu L. Liu X. Xu L. Ruan Y. (2014). Epigenetic control of Pollen Ole e1 allergen and extensin family gene expression in *Arabidopsis thaliana* . Acta Physiol. Plant. 36, 2203–2209. doi: 10.1007/s11738-014-1597-6

[B23] Hurni S. Scheuermann D. Krattinger S. G. Kessel B. Wicker T. Herren G.. (2015). The maize disease resistance gene *Htn1* against northern corn leaf blight encodes a wall-associated receptor-like kinase. Proc. Natl. Acad. Sci. U. S. A. 112, 8780–8785. doi: 10.1073/pnas.1502522112, PMID: 26124097 PMC4507197

[B24] Jain P. Dubey H. Singh P. K. Solanke A. U. Sharma T. R. (2019). Deciphering signaling network in broad spectrum near isogenic lines of rice resistant to *Magnaporthe oryzae* . Sci. Rep. 9, 16939. doi: 10.1038/s41598-019-50990-8, PMID: 31729398 PMC6858299

[B25] Kanneganti V. Gupta A. K. (2008). Wall associated kinases from plants-an overview. Physiol. Mol. Bio Plants. 14, 109–118. doi: 10.1007/s12298-008-0010-6, PMID: 23572878 PMC3550657

[B26] Kin D. Langmead B. Salzberg S. L. (2015). HISAT: A fast spliced aligner with low memory requirements. Nat. Methods. 12, 357. doi: 10.1038/nmeth.3317, PMID: 25751142 PMC4655817

[B27] Kohorn B. D. Greed B. E. Mouille G. Verger S. Kohorn S. L. (2021). Effects of Arabidopsis wall associated kinase mutations on *ESMERALDA1* and elicitor induced ROS. PloS One. 16, e0251922. doi: 10.1371/journal.pone.0251922, PMID: 34015001 PMC8136723

[B28] Kurt P. Yagdi K. (2021). Identifying leaf rust resistance gene Lr10 in some bread wheat using simple sequence repeat (SSR) marker. Kafkas University Journal of Faculty of Agriculture. 24, 850–858. doi: 10.5555/20210359599

[B29] Lally D. Ingmire P. Tong H. Y. He Z. H. (2001). Antisense expression of a cell wall-associated protein kinase, *WAK4*, inhibits cell elongation and alters morphology. Plant Cell. 13, 1317–1331. doi: 10.2307/3871298, PMID: 11402163 PMC135583

[B30] Lekklar C. Pongpanich M. Suriya-Arunroj D. Chinpongpanich A. Tsai H. Comai L.. (2019). Genome-wide association study for salinity tolerance at the flowering stage in panel of rice accessions from Thailand. BMC Genomics. 20, 76. doi: 10.1186/s12864-018-5317-2, PMID: 30669971 PMC6343365

[B31] Lerouge P. (2010). Characterization of a putative 3-deoxy-D-mano-2-octulosonic acid (kdo) tranferase gene from *Arabidopsis thaliana* . Glycobiology. 20, 617–628. doi: 10.1093/glycob/cwq011, PMID: 20124190

[B32] Li B. Dewey C. N. (2011). RSEM: accurate transcript quantification from RNA-Seq data with or without a reference genome. BMC Bioinform. 12, 323. doi: 10.1186/1471-2105-12-323, PMID: 21816040 PMC3163565

[B33] Li X. Qi S. F. Meng L. Z. Su P. S. Sun Y. W. Li N.. (2025). Genome-wide identification of the wall-associated kinase gene family and their expression patterns under various abiotic stresses in soybean (*Glycine max* (L.) Merr). Front. Plant Sci. 15, 1511681. doi: 10.3389/fpls.2024.1511681, PMID: 39886685 PMC11779729

[B34] Li Y. Wang X. Li Y. Zhang Y. Gou Z. Qi X.. (2020). Transcriptomic analysis revealed the common and divergent responses of maize seedling leaves to cold and heat stresses. Genes (Basel). 11, 881. doi: 10.3390/genes11080881, PMID: 32756433 PMC7464670

[B35] Lim C. W. Yang S. H. Shin K. H. Lee S. C. Kim S. H. (2015). The *AtLRK10L1.2*, *Arabidopsis* ortholog of wheat *LRK10*, is involved in ABA-mediated signaling and drought resistance. Plant Cell Rep. 34, 447–455. doi: 10.1007/s00299-014-1724-2, PMID: 25533478

[B36] Loutre C. Wicker T. Travella S. Galli P. Scofield S. Fahima T.. (2009). Two different *CC-NBS-LRR* genes are required for Lr10-mediated leaf rust resistance in tetraploid and hexaploid wheat. Plant J. 60, 1043–1054. doi: 10.1111/j.1365-313x.2009.04024.x, PMID: 19769576

[B37] Lynch M. Conery J. S. (2000). The evolutionary fate and consequences of duplicate genes. Science. 290, 1151–1155. doi: 10.1126/science.290.5494.1151, PMID: 11073452

[B38] Ma Y. Wang Z. Humphries J. Ratcliffe J. Bacic A. Johnson K. L.. (2024). Wall-associated kinase like 14 regulates vascular tissue development in *Arabidopsis* and tomato. Plant Sci. 341, 112013. doi: 10.1016/j.plantsci.2024.112013, PMID: 38309474

[B39] Roulin A. Auer P. L. Libault M. Schlueter J. Farmer A. May G.. (2013). The fate of duplicated genes in a polyploid plant genome. Plant J. 73, 143–153. doi: 10.1111/tpj.12026, PMID: 22974547

[B40] Schurack S. Depotter J. Gupta D. K. Thines M. Doehlemann G. (2021). Comparative transcriptome profiling identifies maize line specificity of fungal effectors in the maize: stilago maydis interaction. Plant J. 106, 733–752. doi: 10.1111/tpj.15195, PMID: 33570802

[B41] Shannon P. Markiel A. Ozier O. Baliga N. S. Wang J. T. Ramage D.. (2003). Cytoscape: a software environment for integrated models of biomolecular interaction networks. Genome Res. 13, 2498–2504. doi: 10.1101/gr.1239303, PMID: 14597658 PMC403769

[B42] Shin K. H. Yang S. H. Lee J. Y. Lim C. W. Brown J. W. S. Kim H. H. (2015). Alternative splicing of mini-exons in the Arabidopsis leaf rust receptor-like kinase lrk10 genes affects subcellular localisation. Plant Cell Rep. 34, 495–505. doi: 10.1007/s00299-014-1729-x, PMID: 25510357

[B43] Shiu S. H. Bleecker A. B. (2001). Plant receptor-like kinase gene family: diversity, function, and signaling. Stke. 113, re22. doi: 10.1126/stke.2001.113.re22, PMID: 11752632

[B44] Sivaguru M. Ezaki B. He Z. H. Tong H. Osawa H. Baluska F.. (2003). Aluminum-induced gene expression and protein localization of a cell wall-associated receptor kinase in *Arabidopsis* . Plant Physiol. 132, 2256–2266. doi: 10.1104/pp.103.022129, PMID: 12913180 PMC181309

[B45] Soares A. L. C. Geilfus C. M. Carpentier S. C. (2018). Genotype-specific growth and proteomic responses of maize toward salt stress. Front. Plant Sci. 30. doi: 10.3389/fpls.2018.00661, PMID: 29899749 PMC5989331

[B46] Stelpflug S. C. Sekhon R. S. Vaillancourt B. Hirsch C. N. Buell C. R. Leon N. D.. (2016). An expanded maize gene expression atlas based on RNA sequencing and its use to explore root development. Plant Genome. 9 (1), 1–16. doi: 10.3835/plantgenome2015.04.0025, PMID: 27898762

[B47] Sun Y. Wang D. Shi M. Gong Y. Yin S. Jiao Y.. (2023). Genome-wide identification of actin-depolymerzing fator gene family and their expression partterns under various abiotic stresses in soybean (*Glycine max*). Front. Plant Sci. 14, 1236175. doi: 10.3389/fpls.2023.1236175, PMID: 37575943 PMC10413265

[B48] Tzin V. Fernande-Pozo N. Richter A. Schmelz E. A. Schoettner M. Schäfer M.. (2015). Dynamic maize responses to aphid feeding are revealed by a time series of transcriptomic and metabolomic assays. Plant Physiol. 169, 1727–1743. doi: 10.1104/pp.15.01039, PMID: 26378100 PMC4634079

[B49] Verica J. A. He Z. H. (2002). The cell wall-associated kinase (WAK) and WAK-like kinase gene family. Plant Physiol. 129, 455–459. doi: 10.1104/pp.011028, PMID: 12068092 PMC1540232

[B50] Wagner T. A. Kohorn W. D. B. (2001). Wall-associated kinases are expressed throughout plant development and are required for cell expansion. Plant Cell. 13, 303–318. doi: 10.1105/tpc.13.2.303, PMID: 11226187 PMC102244

[B51] Wang Y. Derr D. C. Chen G. P. Gu Y. Q. (2019a). Orthovenn2: a web server for whole-genome comparison and annotation of orthologous clusters across multiple species. Nuclc Acids Res. 43, W52–W58. doi: 10.1093/nar/gkv487, PMID: 31053848 PMC6602458

[B52] Wang D. Du M. Lyu P. Li J. Meng H. Liu X.. (2024a). Functional characterization of the soybean Glycine max actin depolymenrization factor *GmADF13* for plant resistance to drought stress. Plants. 13, 1651. doi: 10.3390/plants13121651, PMID: 38931083 PMC11207668

[B53] Wang S. Meng Y. Ding F. Yang K. Wang C. Zhang H.. (2024b). Comparative analysis of TPR gene family in *Cuccurbitaceae* and expression profiling under abiotic stress in *Cucumis melo* L. Horticulturae. 10, 83. doi: 10.3390/horticulturae10010083

[B54] Wang H. Niu H. H. Liang M. M. Zhai Y. F. Huang W. Ding Q.. (2019b). A wall-associated kinase gene *CaWAKL20* from pepper negatively modulates plant thermotolerance by reducing the expression of ABA-responsive genes. Front. Plant Sci. 10. doi: 10.3389/fpls.2019.00591, PMID: 31156664 PMC6528620

[B55] Wasternack C. Hause B. (2013). Jasmonates: biosynthesis, perception, signal transduction and action in plant stress response, growth and development. *An update to the 2007 review in Annals of Botany* . Ann. Botany. 111, 1021–1058. doi: 10.1093/aob/mct067, PMID: 23558912 PMC3662512

[B56] Whyte T. D. Sipahi H. (2024). Bioinformatic and expression analyses of the wall-associated kinase genes under high-temperature stress in sorghum. Physiol. Plant. 176, e14125. doi: 10.1111/ppl.14125

[B57] Xia X. B. Zhang X. Zhang Y. C. Wang L. R. An Q. Tu Q.. (2022). Characterization of the WAK gene family reveals genes for FHB resistance in bread wheat (*Triticum aestivum* L.). Int. J. Mol. Sci. 23, 7157. doi: 10.3390/ijms23137157, PMID: 35806165 PMC9266398

[B58] Yang M. Han Y. Z. HABAIKE A. Y. J. Wang C. L. (2017). Study of RLK6, one member of leucine-rich repeat receptor-like kinases *(LRR-RLKs*) subfamily genes, on process of flowering in *Arabidopsis* . J. Nucl. Agric. Sci. 31, 0654–0662. doi: 10.11869/j.issn.100-8551.2017.04.0654

[B59] Yang J. Xie M. Wang X. Wang G. Zhang Y. Li Z.. (2021). Identification of cell wall-associated kinases as important regulators involved in *Gossypium hirsutum* resistance to *Verticillium dahliae* . BMC Plant Biol. 21, 220. doi: 10.1186/s12870-021-02992-w, PMID: 33992078 PMC8122570

[B60] Yao X. C. Humphries J. Johnson K. L. Chen J. H. Ma Y. X. (2025). Function of WAKs in regulating cell wall development and responses to abiotic stress. Plants. 14, 343. doi: 10.3390/plants14030343, PMID: 39942905 PMC11820136

[B61] Yin X. Y. Hou X. W. (2017). Role of *OsWAK124*, a rice wall-associated kinase, in response to environmental heavy metal stresses. Pak J. Bot. 49, 1255–1261.

[B62] Yu Y. Shi J. Li X. Liu J. Geng Q. Shi H.. (2018). Transcriptome analysis reveals the molecular mechanisms of the defense response to gray leaf spot disease in maize. BMC Genomics. 19, 1–17. doi: 10.1186/s12864-018-5072-4, PMID: 30305015 PMC6180411

[B63] Yu F. Tan Z. Fang T. Tang K. Liang K. Qiu F. (2020). A comprehensive transcriptomics analysis reveals long non-coding RNA to be involved in the key metabolic pathway in response to waterlogging stress in maize. Genes (Basel). 11, 267. doi: 10.3390/genes11030267, PMID: 32121334 PMC7140884

[B64] Yu Q. Zhang Y. C. Liu T. Y. Wang L. Liu Y. Yu S. W.. (2025). Genome-wide characterization of cysteine-rich receptor-like kinase (CRK) gene family in rice and OsCRK26 functional analysis in response to drought stress. Plant Stress. 15, 100733. doi: 10.1016/j.stress.2024.100733

[B65] Zhang N. (2016). ZmWAK-mediated head smut resistance. [PhD thesis]. Beijing, China: Chinese Agricultural University. doi: 10.27628/d.cnki.gzndu.2016.000080

[B66] Zhang S. Chen C. Li L. Meng L. Singh J. Jiang N.. (2005). Evolutionary expansion, gene structure, and expression of the rice wall-associated kinase gene family. Plant Physiol. 139, 1107–1124. doi: 10.1104/pp.105.069005, PMID: 16286450 PMC1283751

[B67] Zhang F. Huang L. Y. Wang W. S. Zhao X. Q. Zhu L. H. Fu B. Y.. (2012). Genome-wide gene expression profiling of introgressed indica rice alleles associated with seeding cold tolerance improvement in a japonica rice background. BMC Genomics. 13, 461. doi: 10.1186/1471-2164-13-461, PMID: 22953761 PMC3526417

[B68] Zhang Z. Ma W. Ren Z. Wang X. Zhao J. Pei X.. (2021). Characterization and expression analysis of wall-associated kinase (WAK) and WAK-like family in cotton. Int. J. Biol. Macromol. 187, 867–879. doi: 10.1016/j.ijbiomac.2021.07163, PMID: 34339786

[B69] Zhang Z. Q. Ma W. Y. Wang H. J. Ren Z. Y. Liu Y. G. He K. L.. (2025). Characterization of the wall-associated kinase (WAK) gene family in Gossypium barbadense reveals the positive role of *GbWAK5* in salt tolerance. Plant Cell Rep. 44, 1–18. doi: 10.1007/s00299-024-03407-4, PMID: 39738693

[B70] Zhang Q. Q. Xu Q. Y. Zhang N. Zhang T. Xing Y. X. Fan Z.. (2024). A maize *WAK-SnRK12-WRKY* module regulates nutrient availability to defend against head smut disease. Mol. Plant. 17, 1654–1671. doi: 10.1016/j.molp.2024.09.013, PMID: 39360383

[B71] Zhong C. Li W. Zhang X. Zhang D. Wen Z. Song W.. (2025). A cell wall-associated kinase phosphorylates NLR immune receptor to negatively regulate resistosome formation. Nat. Plants. 11, 561–579. doi: 10.1038/s41477-025-01949-3, PMID: 40119183

[B72] Zhou W. F. (2022). Effects of *OsPID* gene overexpression on rice morphology and physiology and its role in low phosphprus response. [Master’s thesis]. (Yangzhou, China: Yangzhou University). doi: 10.27441/d.cnki.gyzdu.2022.000646

[B73] Zuo N. Bai W. Wei W. Q. Yuan T. L. Zhang D. Wang Y. Z.. (2022). Fungal CFEM effectors negatively regulate a maize wall-associated kinase by interacting with its alternatively spliced variant to dampen resistance. Cell Rep. 41, 111877. doi: 10.1016/j.celrep.2022.111877, PMID: 36577386

[B74] Zuo W. L. Chao Q. Zhang N. Ye J. R. Tan G. Q. Li B. L.. (2015). A maize wall-associated kinase confers quantitative resistance to head smut. Nat. Genet. 47, 151–157. doi: 10.1038/ng.3170, PMID: 25531751

