# Supplementary figures and images for: Roles of maize WAK gene family in responses to abiotic and biotic stresses, and hormonal treatments
Source: Front Plant Sci. 2025 Sep 16;16:1652811. doi: 10.3389/fpls.2025.1652811 (PMC12479429; doi:10.3389/fpls.2025.1652811)

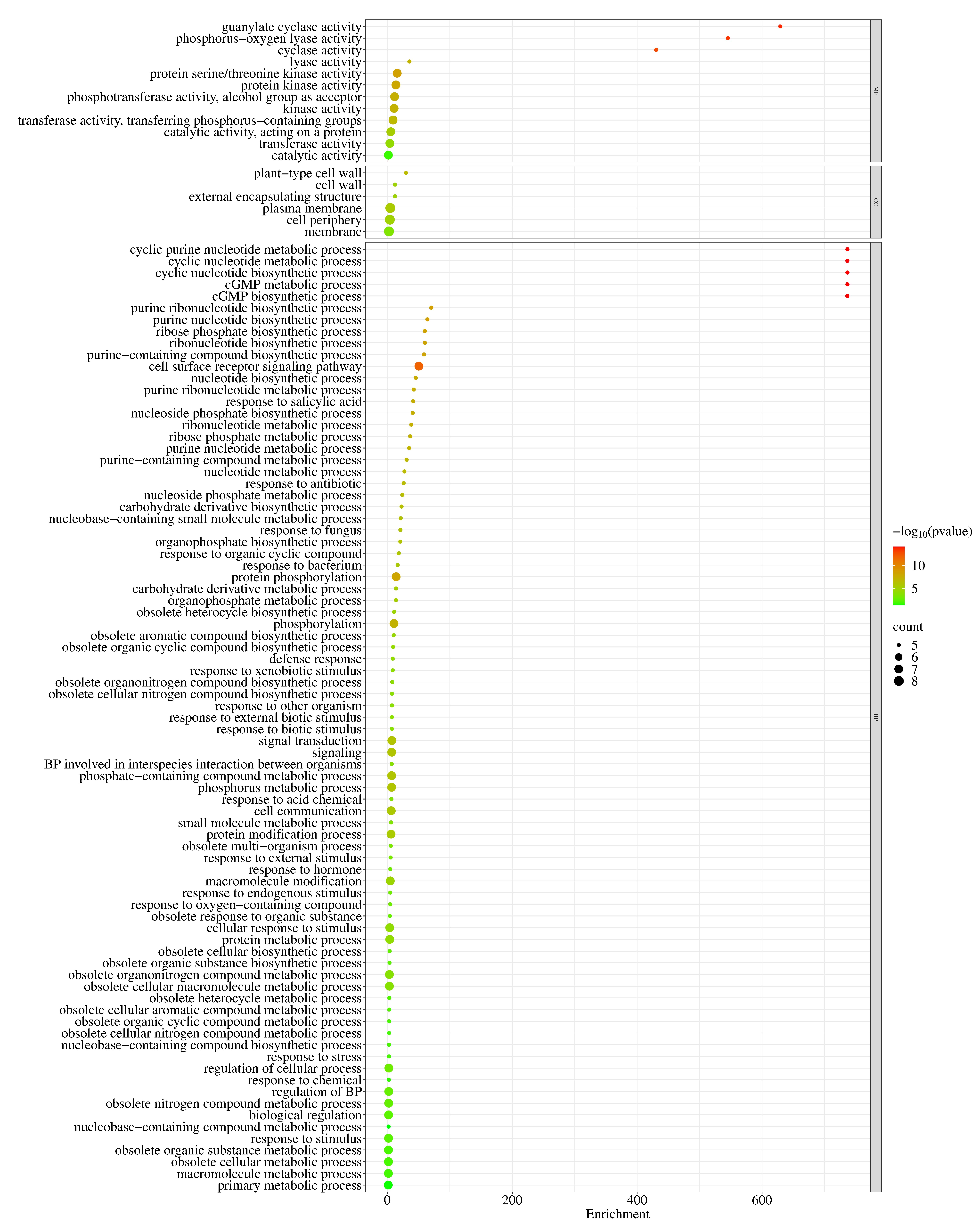

Supplement: Supplementary Figure 1 — GO annotation analysis of ZmWAKs in maize genome. MF indicates molecular function. CC indicates cellular component. BP indicates biological process. Red dot plots indicate the most genes involved in that process, while small green plots indicate the least genes involved. [file Image1.jpeg]

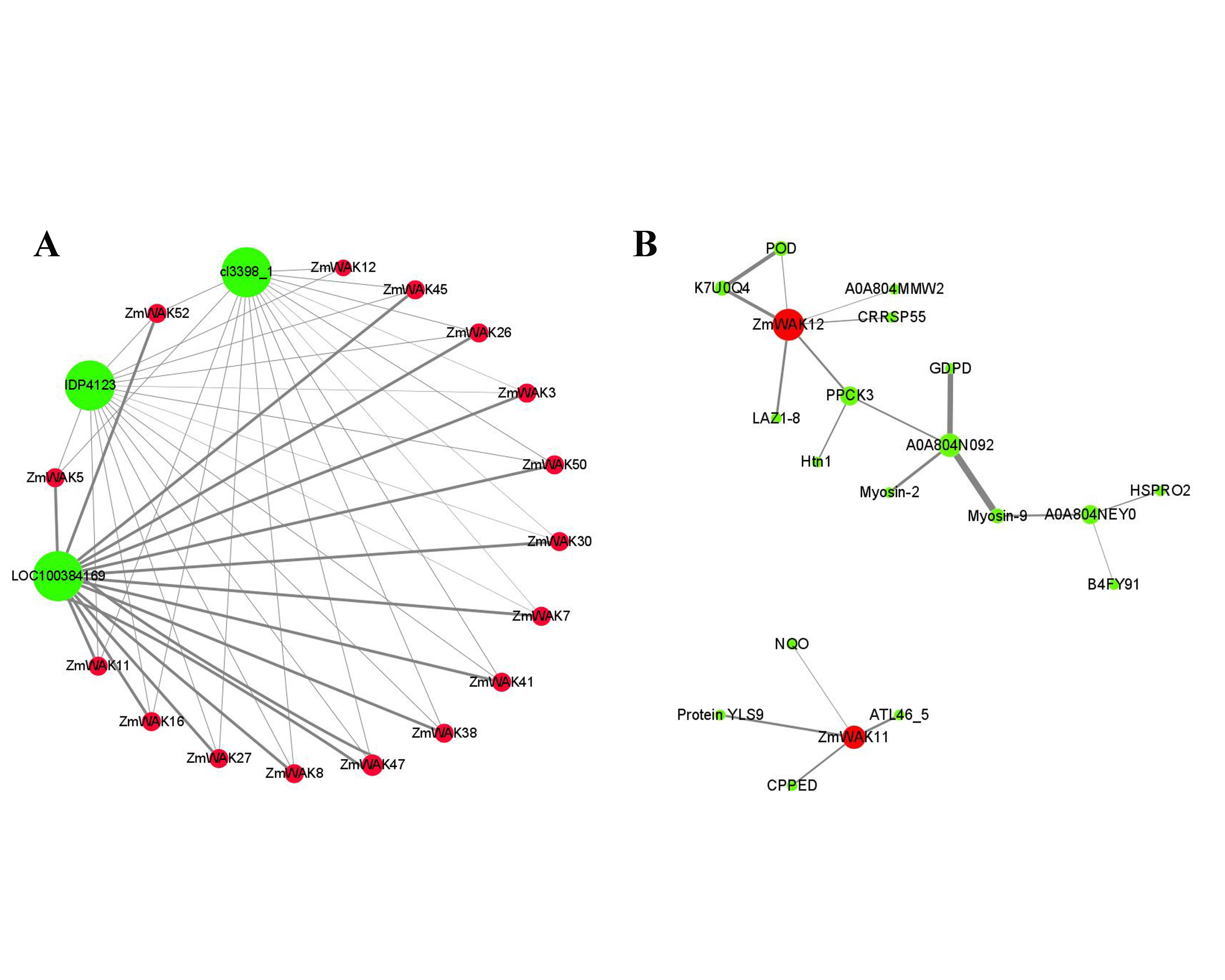

Supplement: Supplementary Figure 3 — The function interaction network of maize WAK proteins. (A) The maize WAK protein interaction network constructed via homology modeling based on Arabidopsis thaliana proteins. (B) The maize WAK protein interaction network constructed via homology modeling based on rice proteins. [file Image3.jpeg]
